# A novel human induced pluripotent stem cell blood-brain barrier model: Applicability to study antibody-triggered receptor-mediated transcytosis

**DOI:** 10.1038/s41598-018-19522-8

**Published:** 2018-01-30

**Authors:** Maria Ribecco-Lutkiewicz, Caroline Sodja, Julie Haukenfrers, Arsalan S. Haqqani, Dao Ly, Peter Zachar, Ewa Baumann, Marguerite Ball, Jez Huang, Marina Rukhlova, Marzia Martina, Qing Liu, Danica Stanimirovic, Anna Jezierski, Mahmud Bani-Yaghoub

**Affiliations:** Human Health Therapeutics Portfolio, National Research Council of Canada, Ottawa, ON K1A 0R6 Canada

## Abstract

We have developed a renewable, scalable and transgene free human blood-brain barrier model, composed of brain endothelial cells (BECs), generated from human amniotic fluid derived induced pluripotent stem cells (AF-iPSC), which can also give rise to syngeneic neural cells of the neurovascular unit. These AF-iPSC-derived BECs (i-BEC) exhibited high transendothelial electrical resistance (up to 1500 Ω cm^2^) inducible by astrocyte-derived molecular cues and retinoic acid treatment, polarized expression of functional efflux transporters and receptor mediated transcytosis triggered by antibodies against specific receptors. *In vitro* human BBB models enable pre-clinical screening of central nervous system (CNS)-targeting drugs and are of particular importance for assessing species-specific/selective transport mechanisms. This i-BEC human BBB model discriminates species-selective antibody- mediated transcytosis mechanisms, is predictive of *in vivo* CNS exposure of rodent cross-reactive antibodies and can be implemented into pre-clinical CNS drug discovery and development processes.

## Introduction

Central nervous system (CNS) drug development is hindered by high clinical attrition rates^1,2^. The complex physiology of the human brain, the difficulty in achieving sufficient drug concentrations in the brain^3^ and inadequate animal models of human CNS pathology are key underlying causes. The development of translational and predictive models for assessing blood-brain barrier (BBB) delivery has become an important requirement in pre-clinical testing of CNS-targeting therapeutics.

The BBB is composed of specialized brain microvascular endothelial cells (BECs) that form a barrier between the bloodstream and the CNS^4^. This diffusion barrier is formed by tight junctions between BECs, which result in a high transendothelial electrical resistance (TEER). In addition to the physical paracellular barrier, the BBB endothelium is enriched with a battery of polarized efflux transporters, that eliminate substrate-drugs from the brain, as well as specialized BBB influx carriers that allow the selective, energy-dependent transport of essential nutrients such as amino acids, carbohydrates and small peptides into the brain^5,6^. The BBB is maintained and regulated by a complex crosstalk between BECs and cells of the neurovascular unit (pericytes, astrocytes, microglia and neurons), which work in concert to ensure proper brain homeostasis and function^7^.

The BBB also hinders the delivery of many potentially important diagnostic and therapeutic agents to the brain. Very few synthetic molecules (highly lipophilic or hydrophobic molecules with a molecular mass below 400–500 Da) and biologics delivered intravenously, can cross the BBB sufficiently to produce a pharmacological effect^8^. In a study evaluating more than 7 000 drug compounds, only 5% could cross the BBB and produce a pharmacological response in the CNS^9,10^.

*In vitro* BBB models have been developed to aid in the pre-clinical evaluation and selection of prospective BBB-permeant drugs and are widely implemented in the biopharmaceutical industry. Most *in vitro* BBB models are constructed using primary BECs isolated from animal brain tissues (reviewed in^11,12^); however, recent discoveries of significant species differences in the abundance and function of key BBB transporters^13–18^ have highlighted the need for the development of human BBB models. Such human BBB models aim to improve translational predictability and ultimately increase the clinical success of CNS pipelines. To date, human BEC sources for BBB models have been derived either from primary biopsied brain tissue^13,14^ or immortalized cell lines^15–18^. Although both models have contributed valuable insights into the cellular and molecular biology of this specialized endothelium, they have limitations as models for drug screening and transport evaluation. Primary BECs are limited in terms of availability of human tissues, scalability and rapid loss of BEC phenotype in culture^19^; immortalized BECs are readily scalable but often suffer from suboptimal barrier properties in culture such as low baseline TEER values and discontinuous tight junction protein expression^18^. Recently, stem cell sources have demonstrated a substantial advantage over other BEC sources for BBB modeling given their human origin, stability, scalability, self-renewal and potential to generate syngeneic cellular components of the neurovascular unit^20–22^. *In vitro* BBB models have been developed from human adult stem cells, specifically human endothelial progenitor cells^23^ and human hematopoietic stem/progenitor cells^24^ as well as from human embryonic stem cells and induced pluripotent stem cells (iPSCs)^25,26^ and were shown to possess many BBB-properties such as high TEER, expression of BEC-specific transporters and *in vitro-in vivo* predictability of transport for a subset of synthetic compounds^24,26^. Despite this significant progress, stem-cell derived BBB models require cell surface marker enrichment and/or co-differentiation and purification steps to yield a pure population of specialized brain endothelial cells (BECs)^23,24,26^.

Here we describe an improved direct monolayer differentiation protocol for the generation of induced BECs (i-BEC), as well as syngeneic neurons and astrocytes, from amniotic fluid-derived induced pluripotent stem cells (AF-iPSCs). The i-BECs exhibit a BBB-specific gene/protein expression profile, high inducible TEER values and functional polarized BBB transport. The i-BECs are used to assemble an *in vitro* BBB model which demonstrates correlative *in vitro-in vivo* receptor mediated transcytosis using species cross-reactive BBB-crossing antibodies. This is the first stem cell derived human BBB model that is extensively characterized for receptor-mediated transport and its utility in evaluating antibody-based BBB carriers.

## Results

### Generation of iPSCs from amniotic fluid cells

**Figure 1:**
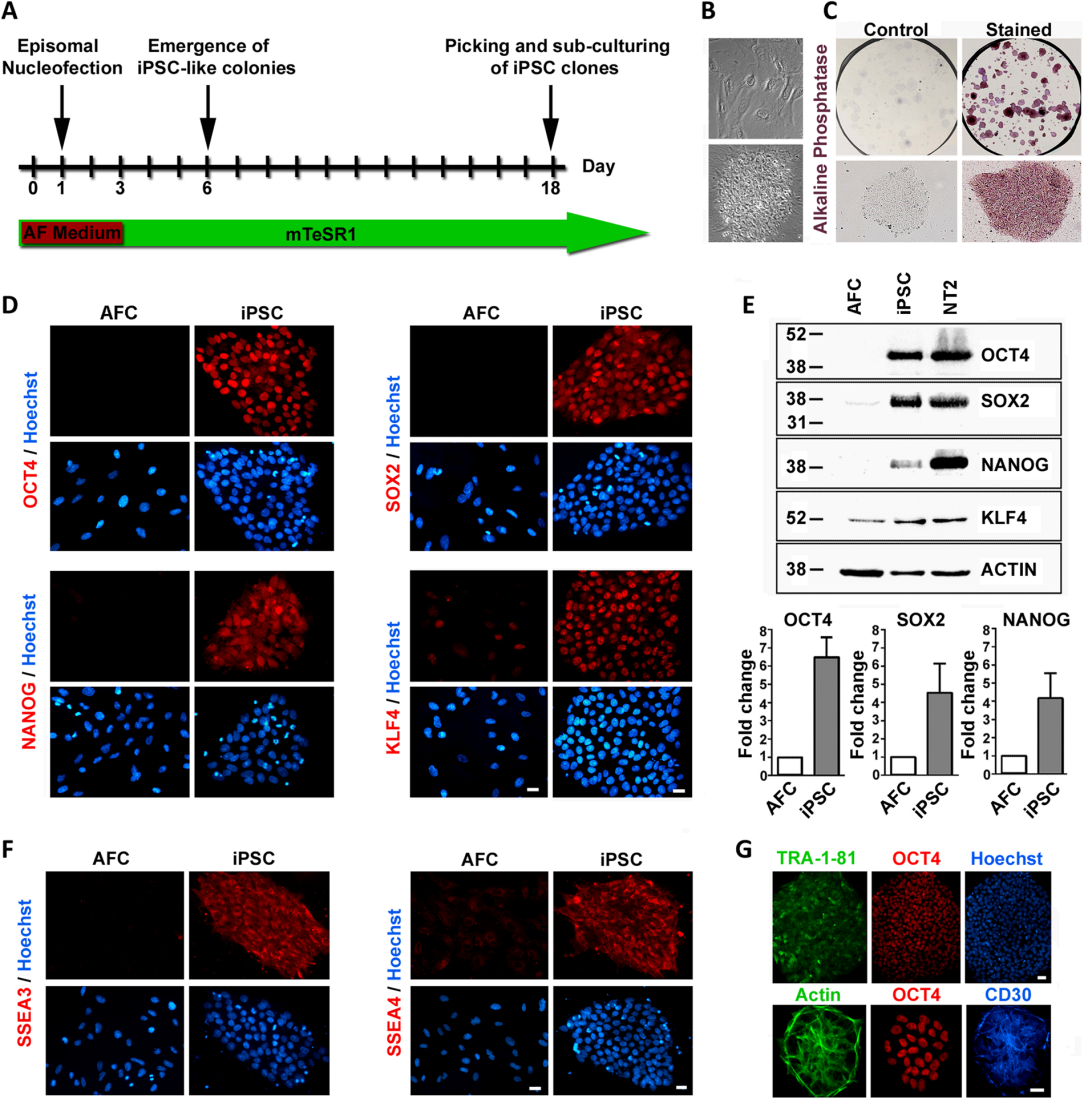
Generation and characterization of induced pluripotent stem cells (iPSCs) derived from amniotic fluid cells (AFCs). (**A**) Schematic of AF-derived iPSC generation time course. iPSC-like colonies began to emerge at day 8 post-transfection and iPSC colonies were picked and sub-cultured at day 18. (**B**) Phase contrast image of parental AFCs (top panel) and the established AF-derived iPSC colony (bottom panel) which shows a typical embryonic stem cell-like morphology in feeder-free conditions. (**C**) Alkaline phosphatase staining of established iPSC colonies shows positive alkaline phosphatase activity in mTeSR1 maintenance conditions. (**D**) Immunofluorescence staining demonstrating the expression of pluripotency-associated markers OCT4, NANOG, SOX2, and KLF4 in iPSCs compared to parental AFCs. (**E**) Representative cropped western blot comparing the expression of OCT4, NANOG, SOX2 and KLF4 in AFCs, iPSCs and control NT2 cells. Fold-change increase in expression levels of OCT4, SOX2 and NANOG in iPSCs relative to AFCs is shown (mean ± SEM). (**F**) Immunofluorescence staining of human embryonic stem cell-specific surface antigens SSEA3 and SSEA4 in iPSCs compared to AFCs. (**G**) Confirmation of fully reprogrammed state of iPSCs by the concomitant expression of TRA-1-81, OCT4 and CD30. Nuclei counterstained with Hoechst. Scale bar, 20 µm and 40 µm (top panel 1 G).

Induced pluripotent stem cells (iPSCs) were generated from human amniotic fluid derived cells (AF-iPSCs) using non-integrating episomal vectors in feeder-free conditions. AF cells were reprogrammed using the oriP/EBNA1 episomal vectors encoding OCT4, SOX2, c-Myc, KLF4, NANOG and LIN28^27^. Figure 1A illustrates the reprogramming kinetics of iPSC generation. iPSC-like colonies began to appear approximately 6 days post-transfection and iPSC colonies were mechanically picked and passaged at day 18. This quick emergence of iPSC-like colonies, coupled with a relatively high reprogramming efficiency (0.05%) for episomal vectors in our cells, is consistent with previous studies highlighting the increased reprogramming efficiency of fetal-derived AF cells in generating iPSC lines^28–30^. The iPSC colonies were distinguishable by their characteristic human embryonic stem-cell morphology, compact colonies with high-nucleus-to-cytoplasm ratio (Fig. 1B) and alkaline phosphatase activity (Fig. 1C). The established iPSC colonies maintained a normal karyotype in culture (Supplementary Fig. 1A) and showed distinct OCT4, SOX2, NANOG and KLF4 nuclear staining (Fig. 1D) with several fold-increase in protein expression over parental AF cells (Fig. 1E). In contrast to the parental AF cells, the OCT4 and NANOG promoters were hypomethylated in the AF-iPSCs and PCR analysis demonstrated the absence of the vector and transgene sequences in the established AF-iPSCs confirming the endogenous expression of reprogramming genes (Supplementary Fig. 1B,C). Compared to the parental AF cells, the AF-iPSCs expressed human embryonic stem cell-specific surface antigens (SSEA) SSEA3 and SSEA4 (Fig. 1F) as well as TRA-1-81 (Fig. 1G), which is detected primarily at later stages of reprogramming^31^. The concomitant expression of the cluster of differentiation 30 (CD30), a specific pluripotency marker shown to distinguish fully reprogrammed iPSCs from other partially reprogrammed derivatives^32^, with OCT4 and TRA-1-81 was also observed (Fig. 1G).

### Differentiation of AF-iPSCs into neurons and astrocytes

**Figure 2:**
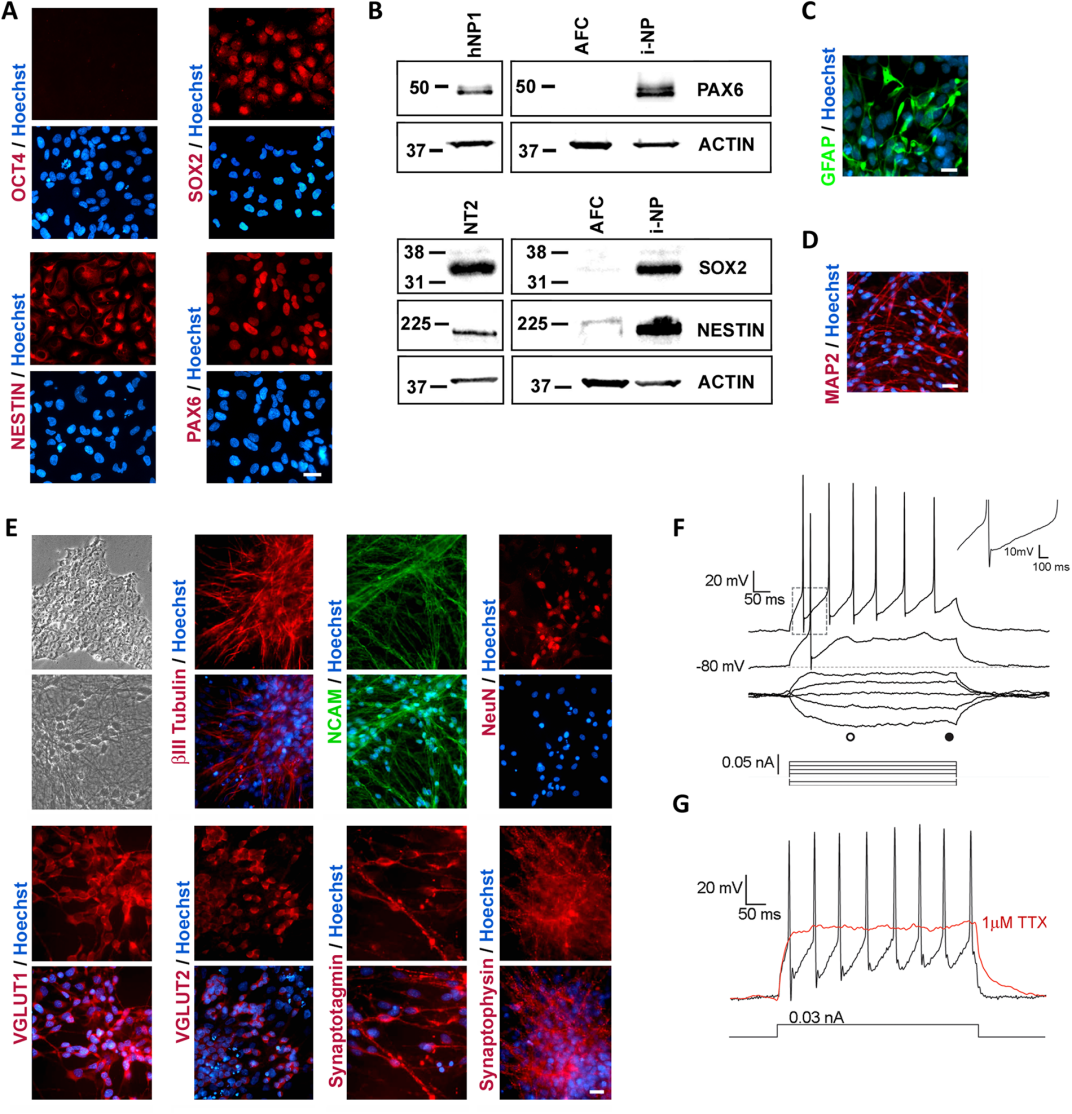
Differentiation of AF-iPSCs into induced neural progenitors (i-NPs) and functional neurons (i-Ns). (**A**) Immunofluorescence staining of i-NPs negative for OCT4 staining and positive for neural progenitor markers SOX2, NESTIN and PAX6. (**B**) Representative cropped western blot confirming increase in expression of PAX6, SOX2 and NESTIN in i-NPs compared to AFCs. Human neural progenitor cells (hNP1) and NT2 cells were used as positive controls. Under specific neural induction conditions (see Methods), the i-NPs gave rise to syngeneic (**C**) GFAP positive astrocytes and (**D**) MAP2 positive neurons. (**E**) Phase contrast images of representative morphology of iPSCs (top panel) in maintenance culture and i-Ns following neuronal differentiation. The mature neurons acquired characteristic neuronal morphology with defined cell bodies and branching neurite extensions (lower panel). Immunofluorescence staining for several neuronal markers including βIII-TUBULIN, NCAM, NeuN, VGLUT1, VGLUT2, SYNAPTOTAGMIN and SYNAPTOPHYSIN expressed in mature i-Ns. (**F**) Electrophysiological properties of i-Ns was assessed using typical voltage responses to a series of current pulses (bottom traces) applied at resting membrane potential (V_m rest_) of -80 mV. Action potentials were evoked by depolarizing current pluses. Top right inset (dotted square) shows magnification of fast and slow after-hyperpolarization (I_hap_) of an evoked action potential. (**G**) Voltage responses to a 0.03 nA current step in absence (black trace) and presence of 1 µM tetrodotoxin (TTX; red trace), a potent sodium blocker which inhibits spiking activity. All recordings were obtained using the whole-cell patch-clamp technique in current clamp mode. Representative traces from individual neurons are shown (n = 8). Nuclei counterstained with Hoechst. Scale bar, 20 µm.

The neural ectodermal lineage differentiation of AF-iPSCs was induced using Stemdiff Neural Induction medium. AF-derived induced neural progenitor (i-NP) cells began to emerge as a morphologically homogenous confluent monolayer negative for OCT4 staining and positive for neural progenitor markers SOX2, NESTIN and PAX6 (Fig. 2A,B). These i-NPs formed neural rosettes and neurospheres in culture and under specific induction conditions (see Methods), these neural rosettes and neurospheres could be differentiated towards syngeneic astrocytes (Fig. 2C) and neurons (Fig. 2D). The purity of the neuronal and astrocytic differentiation is shown in Supplementary Fig. 2. Following 4 weeks in neuronal induction conditions, mature neurons (i-Ns) appeared with characteristic neuronal morphology of defined cell bodies and neurite extensions accompanied by the expression of differentiated neuronal markers βIII-TUBULIN, NCAM, NeuN, glutamate transporters VGLUT1, VGLUT2 and synaptic proteins SYNAPTOTAGMIN and SYNAPTOPHYSIN (Fig. 2E). To demonstrate that the i-Ns express the repertoire of voltage-gated ion channels characteristic of active neurons, the i-Ns were recorded using whole-cell patch-clamp technique in current clamp mode. The i-Ns had an average resting membrane potential (V_m rest_) of −84.21 ± 3.92 mV and an averaged input resistance (R_in_) of 2454.53 ± 470.21 MΩ. The high value of the R_in_ is in accordance with previous reports on iPSC-derived neuronal electrophysiological recordings^33^. Following current injections, the i-Ns exhibited a sustained series of action potentials. These action potentials had an average amplitude of 77.82 ± 8.37 mV and a duration at half amplitude of 2.64 ± 0.38 msec. They also had a mix of fast after-hyperpolarizing and slow after-hyperpolarizing currents (Fig. 2F). The addition of 1 µM tetrodotoxin (TTX), a potent sodium channel blocker, inhibited the generation of action potentials (Fig. 2G) confirming that the i-Ns were functional neurons.

### Differentiation of AF-iPSCs into brain endothelial cells

**Figure 3:**
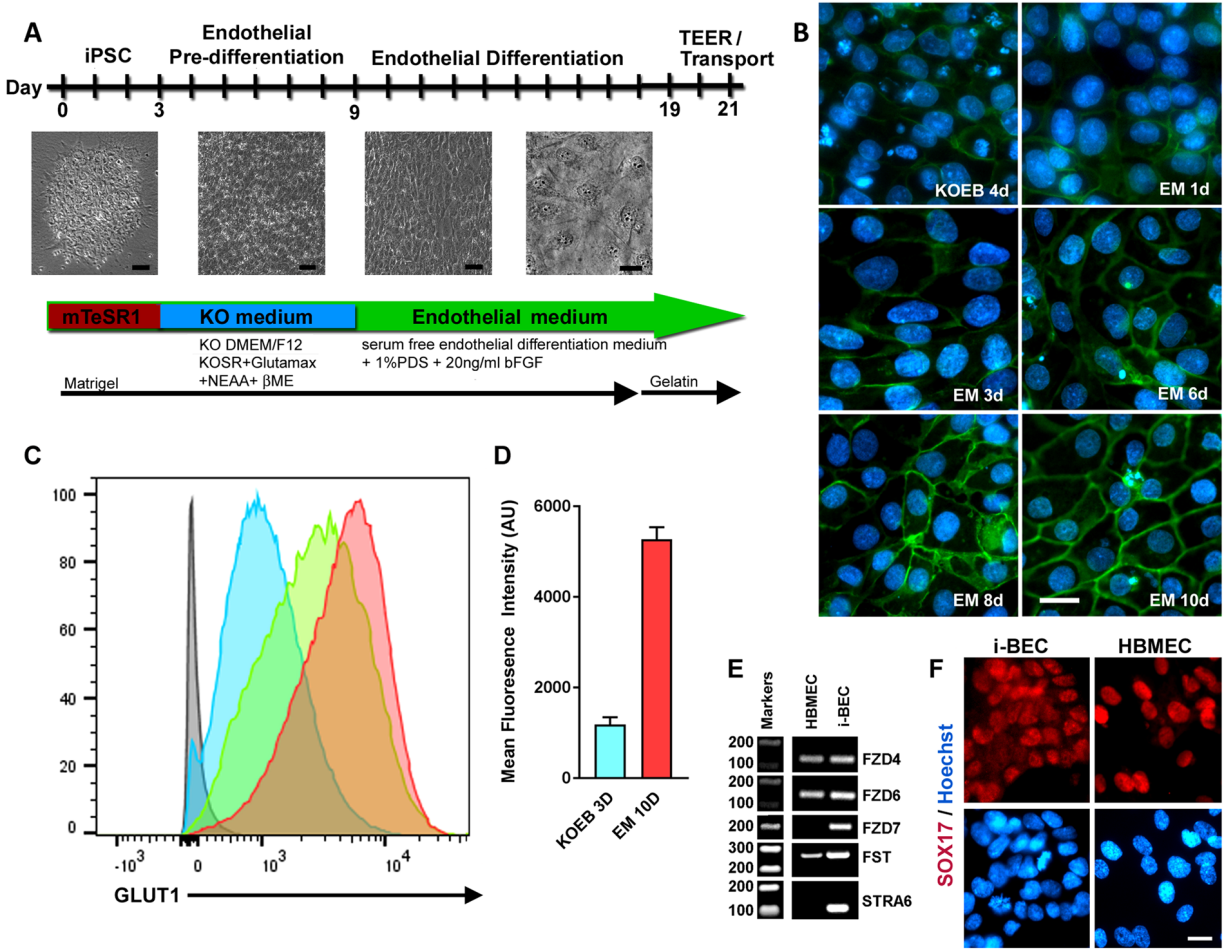
Differentiation of AF-iPSCs into induced brain endothelial cells (i-BECs). (**A**) Schematic of AF- iPSC BEC directed differentiation process depicting the transitional stages of i-BEC monolayer differentiation. (**B**) Representative immunofluorescence depicting temporal GLUT1 expression during pre-differentiation and endothelial differentiation and specification in KOEB and EM medium, respectively. Days (d) in each differentiation medium are shown. (**C**) Flow cytometry histograms depicting the increase in mean fluorescence intensity of GLUT1 expression during different stages of differentiation (3D KOEB, blue; 3D EM, green and 10D EM, red). Grey histogram depicts unstained cells. (**D**) Quantitation of mean fluorescence intensity (arbitrary units, AU) of GLUT1 expression is shown during KOEB 3D and EM 10D differentiation timeframe (mean ± SD, n = 3). Results are representative of at least two independent differentiations. (**E**) Cropped gel electrophoresis of RT-PCR products for transcripts encoding Wnt-β-catenin signaling intermediates involved in BBB specification such as: *FZD4*, *FZD6*, *FZD7*, *FST* and *STRA6* in i-BECs and HBMECs. (**F**) Ubiquitous expression of SOX17, a Wnt-β-catenin downstream signaling target, in i-BECs and HBMECs. Nuclei counterstained with Hoechst. Scale bar, 20 µm.

We specifically set out to differentiate AF-iPSCs into brain endothelial cells (i-BEC) following a two-step differentiation protocol adapted and modified from Lippmann *et al*.^26^ Osmolality of iPSC culture medium can influence iPSC proliferation, survival and differentiation. An optimal osmolality to maintain iPSC pluripotency in culture was found to be approximately 340 mOsm/kg^34^ (osmolality of mTeSR1 medium) which is relatively higher compared to other mammalian cell lines that range from 260 to 320 mOsm/kg^35^. Furthermore, manipulating the osmolality of the iPSC culture medium can also induce differentiation of the iPSCs towards specific germ layer progenitor cells^36^. We found that culturing the AF-iPSCs in low-osmolality KnockOut DMEM/F12 medium (~276 mOsm/kg, KOEB) caused the iPSCs to increase in size and assume a homogenous cobblestone-like morphology as early as 2–3 days in culture (Fig. 3A and Supplementary Fig. 3A). Once the cells formed a uniform monolayer of endothelial-like cells, around 5–7 days in KOEB, the medium was switched to endothelial differentiation medium composed of human serum free endothelial medium (EM) supplemented with 1% platelet-poor plasma derived serum (PDS) and 20 ng/ml bFGF. Following 10 days in EM, the cells acquired a typical cobblestone morphology characteristic of differentiated endothelial cells (Fig. 3A and Supplementary Fig. 3A). To monitor the differentiation process, we examined the temporal expressions of BBB glucose transporter 1 (GLUT1) by immunofluorescence and flow cytometry (Fig. 3B,C). In contrast to previously published protocols^26^, we found that the cells expressed low levels of GLUT1 as early as 3 days in KOEB with a 4-fold increase in GLUT1 mean fluorescence intensity (MFI) observed following 10 days in EM culture (Fig. 3B–D). This degree of homogeneity, at the onset of pre-differentiation (Supplementary Fig. 3A), enabled direct monolayer endothelial cell differentiation and maturation to progress within the same well without the requirement for any co-differentiation or additional purification/enrichment steps. Although GLUT1 is also expressed by epithelial cells (but not neurons and astrocytes), the observed progression of GLUT1 expression in differentiating cultures with the ubiquitous positive staining of the i-BECs for Ulex Europaeus Lectin 1 (ULEX) (Supplementary Fig. 3B), which selectively binds to the l-fucose glycoprotein residues on human brain endothelial cells^37^, confirms endothelial cell specification.

Since the canonical Wnt-β-Catenin signaling pathway has been implicated in BBB specification, microvascular angiogenesis and barrier formation properties of brain endothelial cells^26,38^, we examined transcript expression of Wnt receptors *Frizzled4* (*FZD4*)*, Frizzled6* (*FZD6*) and *Frizzled7* (*FZD7*) and Wnt-downstream target genes *Follistatin* (*FST*) and *STRA6*, which have been shown to be upregulated during the course of BBB differentiation^26^. Specifically, *FZD6* and *STRA6* expression has been shown to be enriched in adult brain endothelial cells compared with lung and liver endothelial cells^38^ and *FZD7* expression has been shown to control paracellular permeability and junction organization^39^. These Wnt receptors and target genes were all highly expressed in the i-BEC in contrast to primary human brain microvascular endothelial cells (HBMEC), which did not express *FDZ7* and *STRA6* (Fig. 3E). SOX17, a downstream transcriptional target of Wnt-β-Catenin, which is robustly expressed in endothelial cells of intracerebral arteries^40,41^, was also expressed in i-BEC cells (Fig. 3F).

**Figure 4:**
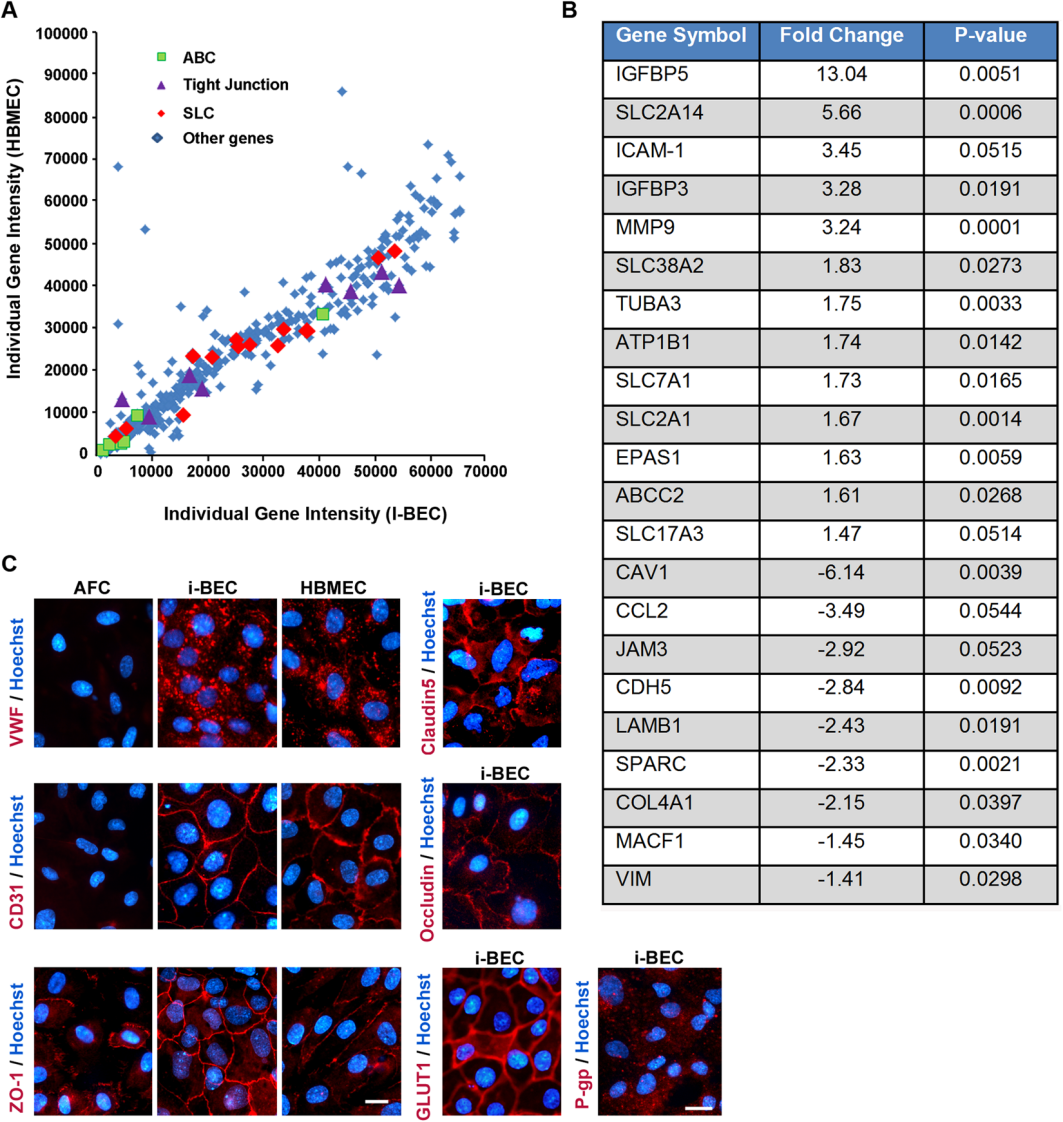
BBB and endothelial specific gene expression in i-BECs. (**A**) A comparative gene microarray analysis comparing the individual gene intensity signals (arbitrary units) of 434- BBB and endothelium-specific genes between HBMEC (y-axis) and i-BECs (x-axis). Scatter plot demonstrates a similar expression profile between both cell types, specifically in ABC transporters (green squares), tight junctions (purple triangles) and SLC transporters (red diamonds) transcripts (Pearson Correlation, r^2^ = 0.86, p = 0.001). (**B**) Table compiling a list of differentially expressed transcripts in i-BECs when compared to HBMECs. Statistical significant was determined using Student’s T-Test, (*p ≤ 0.05) and a 1.35 fold change in ratio was used to generate the gene list. (**C**) Representative immunofluorescence staining of i-BECs for vWF, Claudin-5, CD31 (PECAM-1), Occludin, ZO-1, GLUT1 and P-gp. HBMECs were used as a positive control. All characterizations were carried out at day 21 of EM differentiation. Nuclei counterstained with Hoechst. Scale bar, 20 µm.

To ascertain whether the i-BECs mimic the transcriptome of primary HBMECs, we performed a comparative gene microarray analysis using a 434-gene array containing BBB- and endothelium-specific genes. We found similar gene expression patterns between both endothelial cell types, with approximately 93% of the genes in the BBB microarray being expressed by the i-BECs (Fig. 4A). Of note, transcripts upregulated in i-BECs (compared to primary HBMEC) include several SLC transporters, ABC transporters as well as transcripts of secreted IGF1-binding proteins. Moderate down-regulation of cytoskeletal, ECM and adherens junction genes were also observed with the most down-regulated transcript being *Caveolin-1*. The differentially expressed genes are shown in Fig. 4B and the full individual gene intensity microarray data set is included in Supplementary Table 4. The expression of a subset of these genes was further validated by RT-PCR (Supplementary Fig. 4) and/or immunofluorescence microscopy (Fig. 4C and Supplementary Fig. 5). Similar to primary HBMEC, i-BECs expressed transcripts and/or proteins characteristic of endothelial cells including von Willbrand factor (vWf) and PECAM-1 (CD31), as well as those specific for brain endothelial cells including tight junction proteins *Claudin-5, Occludin, Zonula Occludens-1* (*ZO-1*), solute carrier (*SLC*) transporters enriched at the BBB such as the glucose transporter *Glut-1* (*SLC2A1*) and *large neutral amino acid transporter-1* (*SLC7A5*). ATP-binding cassette (ABC) transporters that mediate efflux activity at the BBB including *p-glycoprotein* (*P-gp*, *ABCB1*), *breast cancer resistant protein* (*ABCG2*) as well as members of the multidrug resistance protein (MRP) family *ABCC1*(*MRP1*)*, ABCC2* (*MRP2*)*, ABCC3* (*MRP3*)*, ABCC4* (*MRP4*) *and ABCC5* (*MRP5*) are also strongly expressed in i-BECs. Although we did not observe differences in *Claudin-5* gene expression levels, immunostaining for Claudin-5 showed continuous intercellular contacts in i-BECs compared to discontinuous intercellular borders with gaps in the HBMEC cultures (Supplementary Fig. 6).

### Functional BBB characterization of i-BECs

**Figure 5:**
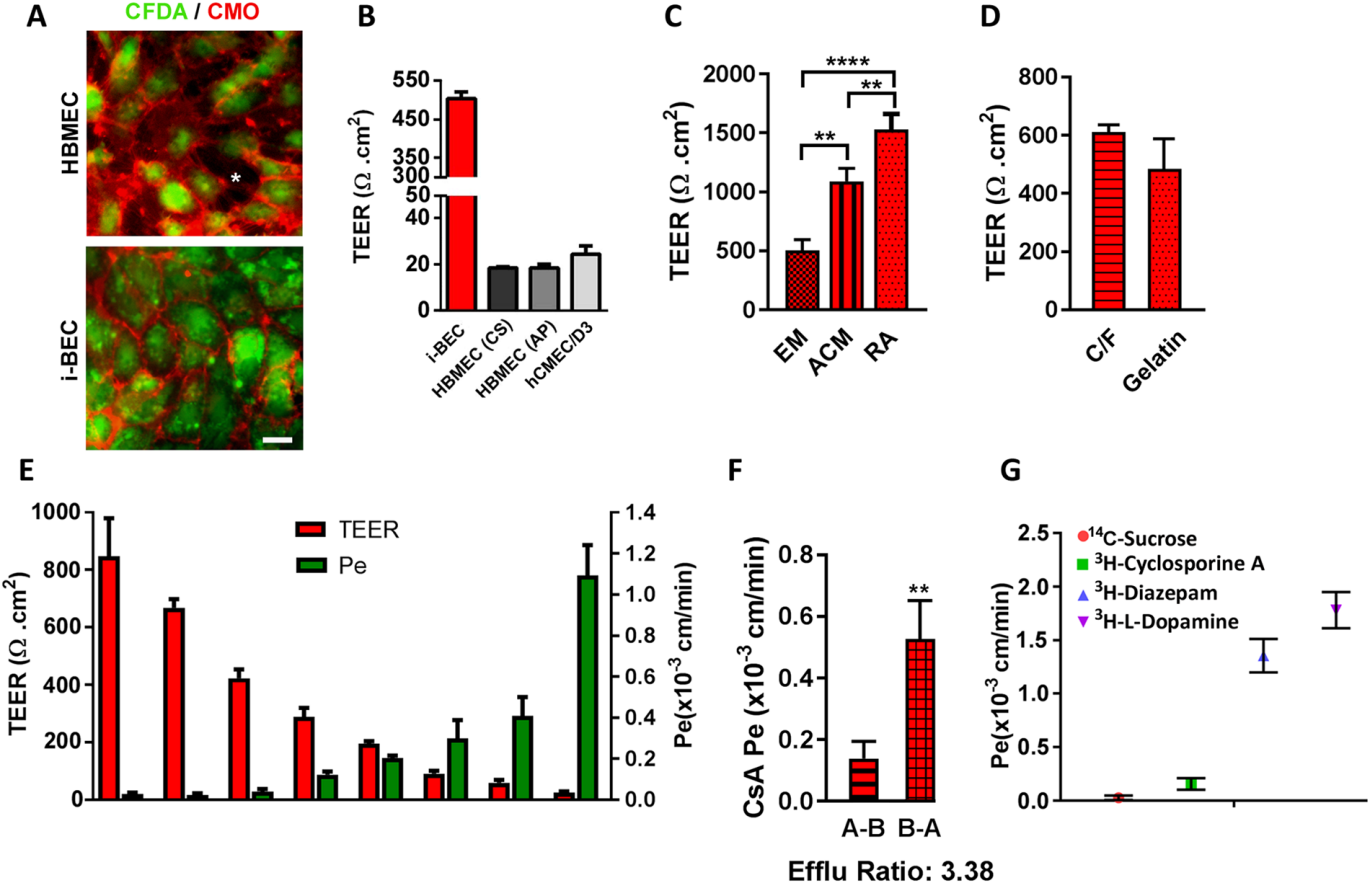
Functional characterization of the i-BECs. (**A**) Live staining of i-BEC and HBMEC cultures with CFDA (cellular viability) and CellMask Orange (plasma membrane) to visually assess the maintenance of continuous tight junctions. Intracellular gaps, indicative of poor BBB integrity, are easily visualized as seen in HBMEC cultures (asterisk, left panel) compared to the tight barrier integrity observed in i-BEC cultures (right panel). (**B**) Transendothelial electrical resistance (TEER, Ωcm^2^) of confluent i-BECs monolayer cultures on gelatin coated Transwell inserts. Higher TEER was observed in the i-BECs compared to HBMECs (CS and AP) as well as immortalized hCMEC/D3 (mean ± SD). (**C**) Enhanced TEER in i-BECs following 24 hr co-culture in astrocyte conditioned medium (ACM) and treatment with 10 µM *all-trans* retinoic acid (RA) compared to EM maintenance culture (mean ± SD, One-Way ANOVA **p < 0.01, ****p < 0.0001). (**D**) Comparison of TEER between i-BECS seeded onto gelatin and collagen/fibronectin (C/F) coated Transwell inserts (mean ± SD). (**E**) Comparison between TEER values (left y-axis) and ^14^C-Sucrose permeability coefficient (P_e_, right y-axis) in i-BECs. Values are mean ± SD for each compound measured in 11 independent inserts from at least 8 biological replicates with varying TEER levels acquired during protocol optimization. The means reflect variability from differentiation to differentiation during protocol optimization steps. (**F**) Permeability values of Cyclosporine A (CsA) from apical to basolateral (A-B) and basolateral to apical (B-A) compartments. Efflux ratio (B-A/A-B) for CsA shown as 3.38. (mean ± SD, Student’s T-test **p < 0.01). (**G**) Functional i-BEC transporter activity was assessed by measuring apical to basolateral permeability (P_e_) using radiolabeled small molecules: ^14^C-Sucrose, ^3^H-Cyclosporine A, ^3^H-Diazepam and ^3^H- l-Dopamine. Values are mean ± SD for each compound measured in at least 3 inserts. Results are representative of at least two independent differentiations. Nuclei counterstained with Hoechst. Scale bar, 20 µm.

The i-BECs were evaluated in a two-compartment *in vitro* Transwell BBB transport assay. Following 10 days in EM culture, the i-BECs were dissociated and seeded at a density of 5.0 × 10^5^ cells/cm^2^ onto gelatin coated 0.9 cm^2^ Transwell polyethylene terephthalate (PET) permeable inserts (1 µm pore size). The establishment of a confluent i-BEC monolayer on the inserts was monitored by a live staining technique using CFDA (a cellular viability dye) and CellMask Orange (CMO), a membrane stain that labels the plasma membrane (Fig. 5A and Supplementary Fig. 7). As shown in Fig. 5A, CFDA/CMO staining showed that the i-BECs formed continuous membrane contacts in confluent cultures, in contrast to the intercellular gaps observed in HBMECs. Transendothelial electrical resistance (TEER), a measurement indicative of barrier impermeability or “tightness” to paracellular diffusion, showed average i-BEC values of 500 Ωcm^2^ (ranging between 300–800 Ωcm^2^ in maintenance culture) 48 hr after seeding onto the inserts (Fig. 5B,E). In comparison, TEER measurements of two commercially available primary HBMECs (HBMEC (from Cell Systems (CS) and AngioProteomie (AP)) and an immortalized HBMEC cell line (hCMEC/D3) ranged from 20–30 Ωcm^2^ (Fig. 5B). TEER measurements following culture of i-BECs in human astrocyte conditioned medium (ACM) increased over 2-fold (1090 ± 112 Ωcm^2^) compared to EM maintenance conditions (503 ± 94 Ωcm^2^) (Fig. 5C) and culture in basal ACM medium containing 1% FBS (779 ± 94 Ωcm^2^), indicating a specific ‘tightening’ response to astrocytic cues. Retinoic acid (RA) treatment, which has been shown to enhance BBB properties and TEER in other iPSC-derived BECs^25,42^, also significantly increased TEER values in the i-BECs (1528.0 ± 132 Ωcm^2^; Fig. 5C). Given the homogeneity of the differentiation protocol, the i-BECs were seeded directly onto gelatin coated inserts without any requirement to stimulate further maturation and purification by sub-culturing onto collagen/fibronectin coated inserts, as described for other human iPSC-derived BEC protocols^26^. No significant changes in TEER were observed following seeding of i-BECs onto Collagen IV/Fibronectin coated inserts, further supporting the purity of the i-BEC culture (Fig. 5D).

i-BEC paracellular permeability (P_e_) for Sucrose, a benchmark of passive transport in *in vitro* models, inversely correlated with TEER values (Fig. 5E) and was similar (P_e_ = 2.8 × 10^−5^ cm/min) to that observed in other human iPSC-derived models (P_e_ = 3.4 × 10^−5^ cm/min)^26^ and substantially lower than that in the hCMEC/D3 line (P_e_ = 1.65 × 10^−3^ cm/min)^18^ and other animal BBB models^43,44^. Sucrose P_e_ did not change with increasing TEER beyond a threshold of 400 Ωcm^2^ (Fig. 5E).

The functional polarization of the transporter activity in maintenance medium was assessed using the model substrate for the efflux transporter P-gp, Cyclosporine A (CsA). The P_e_ values indicated restricted transport of CsA with A-B P_e_ = 0.15 ± 0.05 × 10^−3^ cm/min and B-A P_e_ = 0.53 ± 0.13 × 10^−3^ cm/min with B-A/A-B efflux ratio of 3.38 (Fig. 5F). Diazepam, a lipophilic small molecule with high BBB penetration (P_e_ = 1.36 ± 0.16 × 10^−3^ cm/min) and H- l –Dopamine, a substrate for carrier mediated influx via the organic cation transporter SLC7A5 (LAT-1) (P_e_ = 1.78 ± 0.17 × 10^−3^ cm/min) had the highest *in vitro* permeation (Fig. 5G). This illustrates a 12-fold dynamic range of permeability coefficient among compounds and transporter ligands of various classes (Cyclosporine A/H- l -Dopamine).

### Receptor mediated transcytosis in i-BECs

The focus of this study was the functional characterization of receptor mediated transcytosis (RMT) in the i-BECs, particularly for antibodies raised against specific RMT receptors. RMT uses the vesicular trafficking machinery of BECs to deliver a range of macromolecules to the brain initiated by ligand binding, ligand/receptor internalization, endosomal sorting and subsequent ligand release on the abluminal endothelial surface and receptor recycling back to the luminal membrane^45–48^. Hence, we explored the utility of the i-BEC BBB model for evaluating BBB-crossing antibodies that can be used as molecular Trojan horses for otherwise non-permeable therapeutics.

**Figure 6:**
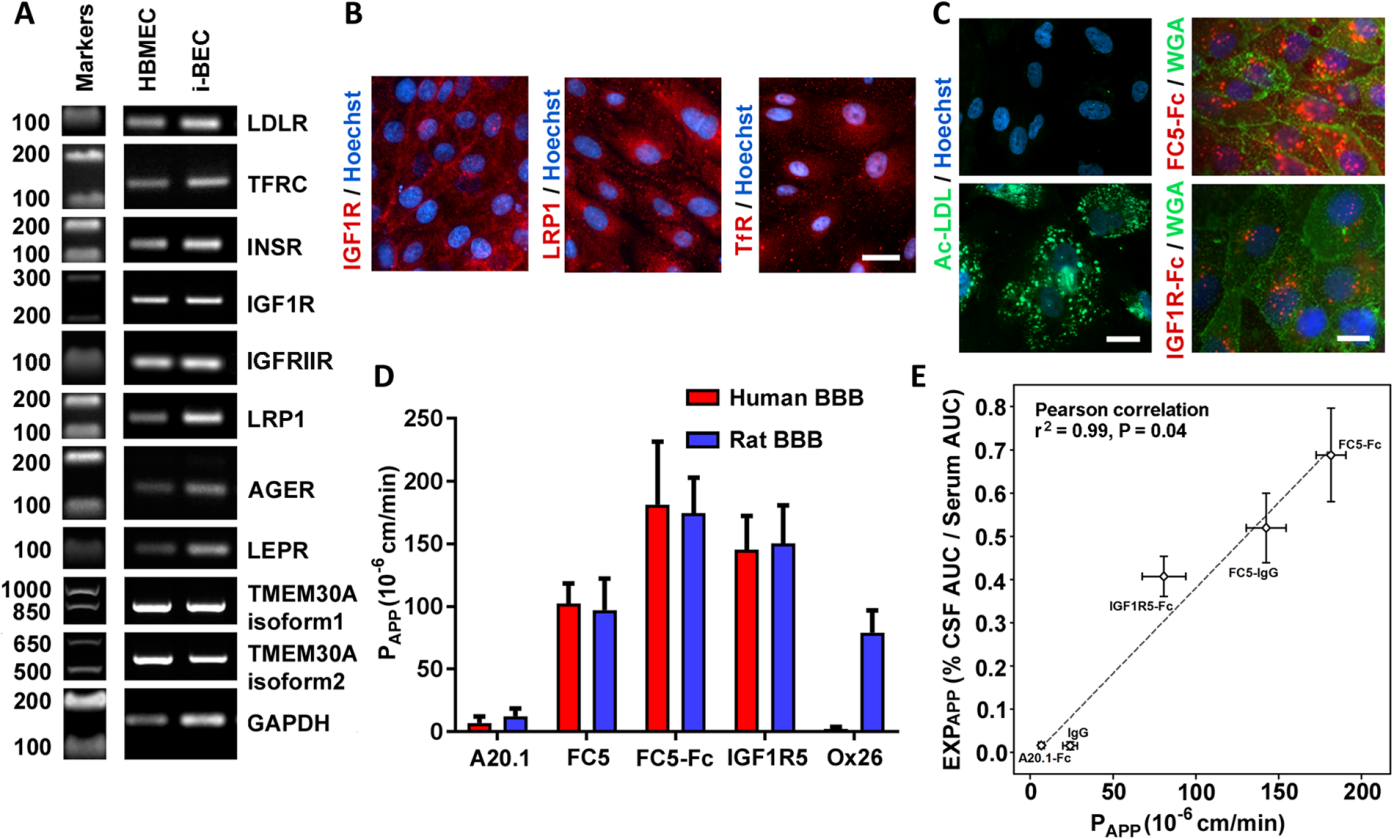
Functional receptor mediated transcytosis in i-BECs. (**A**) RT-PCR detection of receptor mediated transport (RMT) receptor transcripts including *LDLR, TfR, INSR, IGF1R, IGFIIR, LRP1, AGER, LEPR* and *TMEM30A, isoform 1and 2* expressed in i-BECs compared to HBMECs. Cropped gels shown. (**B**) Immunofluorescence staining of i-BECs confirming expression of IGF1R, LRP1 and TfR. Scale bar, 20 µm. (**C**) Receptor mediated endocytosis of acetylated low-density lipoprotein (Ac-LDL), FC5-Fc and IGF1R5-Fc. Plasma membrane stained with Wheat-germ agglutinin (WGA). Scale bar, 20 µm (left panel) and 10 µm (right panel). Nuclei counterstained with Hoechst. (**D**) The *in vitro* apparent permeability coefficient (P_APP_) was assessed for FC5-IgG (FC5), FC5-Fc, IGF1R5 and Ox26 as well as negative control A20.1 antibodies in human i-BEC and rat SV-ARBEC BBB models. (**E**) Correlation of P_APP_ values obtained in the i-BEC BBB model with the apparent CNS exposure (EXP_APP_) for FC5-IgG, FC5-Fc, IGF1R5-Fc and control (A20.1-Fc, IgG) antibodies. EXP_APP_ is derived from simultaneous pharmacokinetic measurements of antibodies in CSF (serial sampling) and in the serum (AUC_CSF_/AUC_serum_) in the rat. The results show a significant correlation (Pearson correlation, r^2^ = 0.96, p = 0.04) among *in vitro* P_APP_ and *in vivo* EXP_APP_ (CNS) for this set of antibodies. Data are mean ± SD of repeated measurements from 3 to 10 *in vitro* BBB model assemblies and from 3 to 6 animals in each group.

The i-BECs express described RMT-mediating receptors, including *Low-Density Lipoprotein Receptor (LDLR), Transferrin Receptor* (*TfR*)*, Insulin* (*INSR*) *and Insulin-like Growth Factor Receptors* (*IGFIR and IGFRIIR*)*, Low-Density Lipoprotein Receptor-related Protein 1* (*LRP1*)*, Receptor for Advanced Glycation End Products* (*AGER*) and *Leptin Receptor* (*LEPR*) as well as the receptor for BBB-crossing antibody *FC5* (*TMEM30A, isoform 1 and 2)* (Fig. 6A,B). The endocytosis triggered by ligand binding to RMT receptors was evaluated using uptake of acetylated low-density lipoprotein (Ac-LDL; ligand to LDLR), as well as antibodies FC5-Fc (binding to TMEM30A) and IGF1R5-Fc (binding to IGF1R). A strong cellular uptake of all three ligands was clearly evident in the i-BECs (Fig. 6C), indicative of functional receptor-mediated endocytosis.

Directional transendothelial transcytosis using a panel of rat-human species cross-reactive camelid single-domain antibodies, (V_H_Hs; 15 kDa) or their fusions with Fc (80 kDa), was compared in the i-BEC and rat immortalized brain endothelial cells (SV-ARBEC^49^) BBB models. The antibodies included a negative control antibody raised against C. difficile toxin A (A20.1; 16 kDa, no mammalian target), FC5, selected by biopanning for its ability to transmigrate the BBB and IGF1R5, raised against an ectodomain of the human IGF1R. An antibody (IgG) specific for rat TfR (Ox26), was also used as a control. The rate of transcytosis of the antibodies (measured by the apparent permeability coefficient, P_APP_) was quantified using highly sensitive multiplexed nanoLC-SRM, as previously described^50–52^. The rat-human cross-reactive antibodies, FC5 and IGF1R5, in V_H_H and Fc-fusion formats, showed similar P_APP_ in both human and rat BBB models, reproducing increased transcytosis rates in bi-valent FC5-Fc format, as previously described^50^ (Fig. 6D). In contrast, rat TfR-specific Ox26 antibody, demonstrated facilitated transcytosis in the rat SV-ARBEC BBB model, and no crossing in the human i-BEC BBB model (Fig. 6D). The P_APP_ of the control, non-binding A20.1 antibody, was 2-fold lower in the i-BEC than the SV-ARBEC model, which correlates with the higher TEER values for i-BEC over that observed in SV-ARBEC model (50–70 Ωcm^2^)^49^ (Fig. 6D).

The P_APP_ values of the IGF1R5 and FC5, as single-domain antibodies and as fusions with human Fc domain observed in the i-BEC model, correlated with the apparent CSF exposure [EXP_APP_] derived from the simultaneous pharmacokinetic measurements of the antibodies in rat CSF and serum [CSF AUC/Serum AUC]. The results show a significant correlation (Pearson correlation, r^2^ = 0.96, p = 0.04) between the *in vitro* P_APP_ and EXP_APP_[CSF] for these antibodies (Fig. 6E), suggesting that the i-BEC model could be used for ranking/predicting *in vivo* rodent exposure of species cross-reactive BBB antibody carriers and potentially de-risking those that are uniquely specific for human antigens.

## Discussion

We have generated iPSCs, from ethically sourced human fetal AF cells, that are transgene-free and derived/maintained in a feeder-free and chemically defined culture system. These AF-iPSCs demonstrate high plasticity and represent a renewable, scalable and reproducible cell source for the derivation of i-BECs as well as other brain cells including neurons and astrocytes. Differentiated i-BECs exhibit a molecular and functional phenotype of HBMECs *in vivo* such as high and inducible TEER, polarization of transporter activity and species-specific properties suitable for use in ‘translational’ screening of early CNS targeting pipelines, especially those targeting receptor-mediated transcytosis.

In an improvement over existing human stem cell/iPSC BEC differentiation protocols^22–24,26^, i-BECs were derived from AF-iPSCs using an efficient and simplified monolayer differentiation protocol without the requirement for cell surface marker enrichment or co-differentiation and purification steps to yield a pure population of BECs. Modulating the osmolality of the pre-differentiation medium, within a range of 270–290 mOsm/kg, increased the homogeneity of the cultures resulting in a higher efficiency of BEC differentiation. Previously published protocols, which employ unconditioned medium (UM) composed of DMEM/Ham’s F12 medium, with an osmolality range of 290–300 mOsm/kg, induced simultaneous co-differentiation of neural and endothelial lineages^26^. This osmolality range for iPSC cultures has been shown to induce mesodermal as well as ectodermal germ layer progenitors from adherent cultures^36^. In our protocol, decreasing the osmolality of the pre-differentiation culture biased the cultures to preferentially differentiate towards BEC. The cells began to acquire homogenous endothelial-like cobblestone morphology with the majority of cells expressing low levels of BBB transporter GLUT1 as early as 2–3 days in KOEB. During the differentiation time course, the MFI of GLUT1 expression increased during the maturation in EM. By comparison, the majority of cells at 3–4 days in UM were neural-like with endothelial-like cells and GLUT1 expression increasing by 6 days in UM^26^. Given the homogeneity of the differentiation in KOEB, no additional sub-culture onto fibronectin/collagen matrix was required to achieve a pure, confluent monolayer of i-BECs. TEER values, averaging 500 Ωcm^2^, were achieved in i-BEC monolayer maintenance cultures in the absence of conditioned medium or hydrocortisone/RA treatments used to increase TEER in other human BBB models^42,53^. This high baseline TEER values translate into a low sucrose permeability (2.8 × 10^−5^ cm/min) similar to that observed in other human iPSC-derived BBB models (3.4 × 10^−5^ cm/min)^26^ and much lower than that observed in hCMEC/D3 (1.65 × 10^−3^ cm/min)^18^. TEER values increased substantially in the presence of human astrocyte conditioned medium (up to 1000 Ωcm^2^) and RA (up to 1500 Ωcm^2^). Recent reports have demonstrated a synergistic effect of RA and NVU co-culture models of pericytes, astrocytes and neurons resulting in increased TEER values ranging from 2500–5000 Ωcm^2^ ^20,22,42,54^. In fact, human iPSC-derived EC co-cultured in the presence of syngeneic pericytes, astrocytes and neurons generated culturally induced-BECs (ciBEC) with BBB specific properties^22^. Although we have shown that TEER is inducible in the i-BEC model, the potential synergies among co-culture with cells from the NVU and chemical (RA) induction remains to be fully examined.

Confirming the fidelity of differentiation, a comparative gene microarray analysis between i-BEC and primary HBMECs (using a 434-gene array containing BBB- and endothelium-specific genes) showed a statistically significant overlap between the two cell types with 93% of the transcripts being similarly expressed. The expression of selected BBB transporters from SLC and ABC families has been confirmed in i-BECs by PCR and functional studies including a polarized transport of the P-gp substrate Cyclosporine A. Transcripts upregulated in i-BECs (compared to primary HBMECs) include several SLC transporters (*SLC2A14*, *SLC38A2*, *SLC37A1*, *SLC2A1* and *SLC17A3*), ABC transporter (*ABCC2*) as well as transcripts of secreted IGF1-binding proteins (*IGFBP5* and *IGFBP3*). Observed down-regulation of cytoskeletal, ECM and adherens junction genes could be attributed to the differences in culture protocols and ECM coating used to culture the i-BEC (Matrigel/gelatin) versus the HBMECs (attachment factor). The most down-regulated transcript in i-BECs was *Caveolin-1* (*Cav-1*), the principal component of caveolae, endocytic vesicles that provide a route for Cav-1 dependent endocytosis and potentially transcytosis. Caveolae are downregulated in mature BECs^6^. Evidence suggests that BBB dysfunction, following injury or disease, is accompanied by increased levels of *Cav-1* expression and increase BBB permeability^55,56^. For example, BECs derived from patients with Huntington’s Disease expressed higher levels of *Cav-1* compared to BECs from healthy donors, suggesting an impaired transcytotic barrier^57^. Similarly, increased bulk-transcytosis of circulatory albumin across the BBB has been observed in mfsda2 knock-out animals which exhibit up-regulation of caveolae in BECs^58^. Since the down-regulation (6-fold) of *Cav-*1 in i-BEC compared to HBMECs was accompanied by >25-fold increase in TEER, lowering of *Cav-1* may be a good biomarker of barrier maturation in cultured BECs. Although we did not observe differences in *Claudin-5* gene expression levels between i-BEC and HBMECs, the organization of intercellular contacts observed by immunostaining for Claudin-5 showed uniform and continuous staining in i-BEC cultures and punctate contacts with intercellular gaps in the HBMEC cultures. These observations are consistent with published reports showing a strong correlation between intercellular Claudin-5 junctional continuity and barrier integrity, which corresponds with the low TEER and paracellular tightness of primary and immortalized HBMECs^20,42^.

Recent studies using LC/MS/MS-based proteomic analyses on BBB tissues showed significant species differences in the expression of BBB transporters^59–62^, necessitating human *in vitro* BBB models for accurate bioavailability predictions of various synthetic compounds. In addition, the human BBB and neuropil cellular platforms are of particular importance for evaluating an emerging pipeline of antibodies against CNS targets, since they are usually raised against species-specific epitopes. To enable access of therapeutic antibodies to their targets in the CNS, they can be re-engineered into bi-specific antibodies, in which the second ‘arm’ functions as the BBB delivery carrier that initiates a receptor-mediated transcytosis (RMT) of the antibody across the BBB. RMT-targeting antibodies such as TfR and Insulin receptor antibodies are species-selective, necessitating the use of ‘surrogate’ molecules in pre-clinical studies. Therefore, human BBB models are an important tool for evaluating BBB carriers and their conjugates with therapeutic payloads that are selective for human receptors. Furthermore, the specific RMT receptor abundance, which can impact the capacity of a delivery system, can be significantly different among species. For example, TfR is 3-fold more abundant in rat compared to human brain vessels^62–64^, underscoring the importance of human models that replicate *in vivo* species differences. Lastly, the human BBB model is of immense value for human specific antibodies which would otherwise give ‘false-negative’ results in the rat model alone.

To explore the utility of the i-BEC model in the evaluation of RMT, we compared a panel of BBB-crossing antibodies, including FC5 (which binds TMEM30A), IGF1R5 (which binds IGF1R) and Ox26 (which binds TfR) in rat SV-ARBEC and human i-BEC models. FC5 and IGF1R5 have been selected for rodent-human cross-reactivity while Ox26 was selective for rat TfR. Whereas both FC5 and IGF1R5 (as either single-domain antibodies or in fusion with Fc domain) showed similar P_APP_ values in both models, OX26 transcytosis was only observed in the rat BBB model. In both models, Fc fusion of FC5 had higher P_APP_ values compared to monomeric FC5, despite being a substantially larger molecule (i.e., 75 kDa vs 15 kDa, respectively), as previously described^50^. As shown in this example, coupling evaluation of RMT-targeting antibodies in rodent and human BBB models can be an important strategy for identifying species cross-reactive and similarly efficient antibody variants that could be used in pre-clinical *in vivo* brain distribution and pharmacokinetic studies. Using the set of species cross-reactive BBB-crossing re-engineered antibodies^50,65^ we confirmed a significant correlation between their transport (P_APP_ values) in the i-BEC model and their *in vivo* CSF exposure measured from serial pharmacokinetic studies.

Herein, we described the development and characterization of a human BBB model *in vitro* (i-BEC) differentiated from iPSCs derived from amniotic fluid cells using a novel, simplified and efficient method. Collectively, the data shown that i-BECs are a valuable tool for investigating drug classes that target human-enriched or specific BEC transporters, receptors and signaling pathways. The described iBEC differentiation strategy can be extended towards generating patient and disease specific BBB models to examine BBB dysfunction with implications for therapeutics and drug delivery^66,67^. Although *in vitro* BBB culture models are a ‘reductionist’ approach to evaluating the ability of compounds to cross the BBB barrier and cannot truly recapitulate the complex anatomical and functional relationships of the NVU or *in vivo* pharmacokinetics, the emergence of human iPSC-derived BBB models represent an important advancement in the pre-clinical evaluation of CNS therapeutics towards improving clinical translation.

## Methods

### Generation of amniotic fluid-derived induced pluripotent stem cells

Amniotic fluid (AF) cells at 26 weeks of gestation (AFC) were obtained from The Ottawa Hospital (Ottawa, Ontario, Canada) following routine amniocentesis. Informed consent was obtained from each woman to use the amniotic fluid for research purposes and all the methods were carried out in accordance with relevant guidelines and regulations as approved by the Ottawa Hospital Research Ethics Board. All experimental protocols using AF cells were performed following the guidelines established and approved by the National Research Council Canada Research Ethics Board. The AF cells were cultured in Dulbecco’s Modified Eagle Medium (DMEM; Life Technologies) supplemented with 20% inactivated fetal bovine serum (FBS; Wisent). To induce reprogramming, 0.5 × 10^6^ AFCs were nucleofected with 1.3 μg of pEP4 E02S EN2K (Addgene-20925), pCEP4 M2L (Addgene-20926) and pEP4 E02S ET2K (Addgene-20927) episomal vectors using the Nucleofector I Device (Amaxa) and plated into a 6-well plate coated with Matrigel hESC qualified matrix (Corning), prepared as per manufacturer’s instructions, containing pre-warmed DMEM + 20% FBS supplemented with 10 µM Y27632 (ROCK Inhibitor, dissolved in DMSO as a 10 mM stock as per manufacturer’s instructions, Stem Cell Technologies). Two days later, the media was switched to mTeSR1 (Stem Cell Technologies) and replaced daily. Once the iPSC colonies were established, the cells were passaged at 80% confluency at a 1:12 ratio approximately every 5 days using ReLeSR (Stem Cell Technologies) onto Matrigel coated plates. The pluripotency and reprogramming efficiency of the established iPSC colonies was confirmed using Alkaline Phosphatase staining (AP, Invitrogen) and StainAlive Dylight 488 Mouse anti-human TRA1-81 Antibody (Stemgent), as per manufacturer’s instructions. Karyotype-G-banding on established iPSCs was carried out at WiCell Research Institute (WiCell Cytogenetics Lab, Wisconsin). All experiments were carried out between passage number 10–40.

### Differentiation of iPSCs into neural progenitor cells

To generate iPSC derived induced neural progenitor cells (i-NPs), iPSCs were dissociated using ReLeSR (Stem Cell Technologies) and passaged at a 1:6 ratio onto Matrigel coated plates in Stemdiff Neural Induction Medium (Stem Cell Technologies) and changed daily, as per manufacturers protocols. The cells were dissociated using Accutase (Stem Cell Technologies) according to manufactures instructions. After 3 weeks in culture, the i-NPs were replated and maintained in Stemdiff Neural Progenitor medium (Stem Cell Technologies) replenished daily.

### Neuronal differentiation of iPSC

To induce neuronal differentiation, i-NPs were dissociated using Accutase (Stem Cell Technologies), resuspended in Neural Progenitor Maintenance BulletKit Medium (NPMM; Lonza) and plated on uncoated 10 cm petri dishes (Fisher Scientific) for neurosphere formation or on Matrigel (Corning) coated plates for adherent cultures. The neurospheres were passaged by being dissociated into small aggregates using Accutase and re-plating onto Matrigel or 10 µg/ml coated poly- l-lysine (PLL, Sigma) coated plates in DMEM/F12 (Life Technologies) supplemented with B27 (Life Technologies) to induce neuronal differentiation. The medium was replaced every other day and mature neurons were observed following 4 weeks of differentiation in B27 containing medium.

### Astrocyte differentiation of iPSC

To induce astrocyte differentiation, i-NPs maintained in StemDiff Neural Progenitor medium (Stem Cell Technologies) were dissociated using Accutase (Stem Cell Technologies) and plated at a 1:6 ratio in poly-l-ornithine PLO (20 µg/ml, Sigma) and laminin (10 µg/ml, Sigma) coated plates in Neural Progenitor medium for one day. The following day, the medium was changed to astrocyte induction medium composed of DMEM/F12 with Glutamax (Stem Cell Technologies), 5% FBS, 20 ng/ml EGF (Life Technologies) which was replaced every other day for four days. At day 5, the medium was changed to complete astrocyte induction medium supplemented with 10 ng/ml CNTF (Sigma) and the medium was changed every other day. From day 8 to 14, the cells were cultured in DMEM/F12 supplemented with Glutamax, 1% FBS and 10 ng/ml CNTF and the medium was replaced every other day.

### Endothelial cell differentiation of iPSCs

Endothelial cell differentiation protocol was adapted from the two-step differentiation protocol by Lippmann *et al*.^26^ In the initial pre-differentiation step, once the iPSCs reached 60–70% confluency the medium was switched from mTeSR1 to low osmolality KOEB medium composed of KnockOut DMEM/F12 medium (~276 mOsm/kg, Gibco) supplemented with 20% KnockOut serum replacement, 1 × Glutamax, 1 × non-essential amino acids and 55 µM β-mercaptoethanol (all from Life Technologies) for 5–7 days. During this time frame, major morphological changes were observed as the cells became bigger and began to assume a cobblestone-like morphology. Once the cells formed a uniform monolayer of endothelial-like cells, the medium was switched to endothelial differentiation medium (EM) composed of human serum free endothelial medium (Life Technologies) supplemented with 1% platelet-poor plasma derived serum (PDS, Alfa-Aesar) and 20 ng/ml bFGF for 9–10 days. While in EM culture, the cells acquired a typical cobblestone morphology characteristic of differentiated endothelial cells (Supplementary Fig. 4). After 9–10 days in EM, the cells were dissociated with Accutase (Stem Cell Technologies) at 37 °C for 10–15 min. Once dissociated, the cells were filtered through a 40 µm sieve to eliminate residual basement membrane and endothelial cell clusters and re-suspended in EM containing 10 µM ROCK Inhibitor. Singularized i-BECs were plated onto 0.5% gelatin coated Transwell inserts as described under the “Preparation of transwell inserts and TEER measurements” section. *All-trans* 10 µM RA (reconstituted in DMSO at a stock concentration of 10 mM, Sigma) was added following seeding of i-BECs onto inserts where indicated. A mixture of collagen IV (80 µg/ml, Sigma) and fibronectin (20 µg/ml, Sigma) coated on Transwell inserts was used where indicated. All i-BEC characterizations were performed at the end of the differentiation process (21 days from iPSCs) following passage onto gelatin coated inserts and coverslips, where appropriate.

### Cell culture

Human primary brain microvascular endothelial cells (HBMECs; Cell Systems) were maintained in CSC medium (Cell Systems) on attachment factor (Cell Systems) pre-coated tissue culture plates and passaged 1:4 using Trypsin-EDTA (Invitrogen). Human primary HBMECs (AngioProteomie) were maintained in EGM-2MV Bulletkit medium (Lonza) on 0.5% gelatin coated tissue culture plates and passaged at a 1:4 split ratio using Trypsin-EDTA. All HBMEC studies were carried out within 3–10 passages. Unless otherwise specified (Fig. 5C), HBMEC from Cell Systems were used as comparative positive controls in the experiments. Immortalized human brain capillary endothelial cells (hCMEC/D3; generous gift from Dr. Pierre-Olivier Couraud, Institute Cochin, Paris, France), were maintained in EBM-2 basal medium (Lonza) supplemented with 2% heat-inactivated FBS (Hyclone) on rat tail collagen I (VWR) coated tissue culture plates^18^. The cells were split using Reagent Pack (Lonza) at a 1:3 ratio and cell passages 20–30 were used for these studies. Immortalized adult rat brain microvascular endothelial cells, SV-ARBEC, were established by SV-40 transfection of primary rat brain micorovascular endothelial cells, isolated from 24–30 days old Sprague–Dawley rats, as previously described^49^. Cells were grown in M199 based feeding media containing: 0.25% Peptone, 0.9% d-glucose, BME Amino Acids, BME Vitamins and 10% FBS. Cells were routinely split 1:20 every week onto rat-tail collagen type I (VWR, 60 μg/ml) coated plates. SV-ARBEC cell passages 79–86 were used for these studies. NT2/D1 (NT2) embryonal carcinoma (EC) cells (ATCC) were maintained in HG-DMEM (Invitrogen) and 10%FBS (Hyclone) replenished every 2 days and passaged at a 1:3 split ratio using Trypsin-EDTA, as previously described^68^. Human neural progenitor cells (hNP, Aruna Biomedical) were cultured on Matrigel (Corning) coated plates maintained in complete neural progenitor medium as supplied in the hNP1 neural progenitor expansion kit (Aruna) and were used between passage 6–9. All primary cells were cultured according to the supplier’s recommendations and within specific passages number limits.

### Astrocyte conditioned media

Human primary astrocytes (NHA, Lonza) were grown to 80% confluency in Astrocyte growth medium (AGM, Lonza). The cells were washed 2 × with HBSS and incubated in 10 ml AGM supplemented with 1% FBS per T75 flask for 72 hr. The astrocyte conditioned medium (ACM) was collected, pooled, filter sterilized and aliquoted for storage at -20 °C. In the transport studies, 1 ml of ACM was added to 1 ml of EM in the bottom compartment of the companion plates 48 hr before measuring TEER. For control experiments, 1 ml of AGM medium supplemented with 1% FBS (ACM basal medium) was added to 1 ml of EM in the bottom compartment in parallel.

### Immunocytochemistry

Cells were grown in 12 well plates on 15 mm round coverslips coated either with Matrigel (iPSC, i-NP, i-BECs) or PLO/laminin (Neurons and Astrocytes) in the respective growth media. For most antigens, the cells were fixed using Genofix (DNA Genotek) whereas 4% PFA (Sigma) was used for certain nuclear antigens, namely PAX6 and SOX17. In the latter case, the cells were permeabilized with 0.2% Triton X-100 (Sigma) in PBS (without Ca^2+/^Mg^2+^) for 20 min, washed and blocked using DAKO Protein Block Serum Free (Agilent) for 20 min at room temperature. Primary antibodies were prepared using the DAKO Antibody Diluent (Agilent), according to the dilutions described in Supplementary Table 1 and coverslips were incubated for 1hr at room temperature in a humidified chamber. Coverslips were then washed three times for a minimum of 5 min with PBS (without Ca^2+/^Mg^2+^) and incubated with secondary antibodies diluted 1:500 in antibody diluent at room temperature for 1hr in the dark. Secondary only PBS controls were performed in parallel for all staining experiments. The coverslips were then washed three times for 5 min with PBS and mounted using DAKO fluorescent mounting medium (Agilent) spiked with 5 µg/ml of Hoechst 33258 (Sigma) to counterstain nuclei. Images were captured using the Axiovert 200 M Microscope (Zeiss). Cells on coverslips were imaged using a 20 × /0.5 Plan Neofluar objective and live cells were imaged using 20 × /0.4 LD Achroplan Korr(DICII) objective. All secondary only control immunofluorescence images are shown in Supplementary Fig. 8.

### RNA extraction and RT-PCR

Total RNA was extracted from cells, using TriReagent RT (Molecular Research Centre), as per manufacturer’s instructions. The RNA was treated with DNase (Turbo DNA-Free Kit, Ambion) to remove any residual DNA contamination and the RNA concentration was quantified using a NanoDrop ND-100 (Thermo Scientific). cDNA was synthesized using 5–10 µg of RNA using Superscript II Reverse Transcriptase (Life Technologies) and AncT primers (Life Technologies) and purified using the QiaQuick PCR purification kit (Qiagen). The Oligreen Assay (Molecular Probes) was used to measure the concentration of cDNA samples. For each RT-PCR reaction, 10 ng of cDNA, 10 pmol/µl primer sets specific for the genes of interest (Supplementary Table 2) and iQ Supermix (BioRad) were used and the PCR reaction was carried out in a PTC-200 DNA Engine Thermal Cycler (MJ Research) by using the following parameters: Initial denaturation step at 94 °C for 3 min, followed by 30 cycles of a denaturation step at 94 °C for 20 sec, annealing at 60 °C for 20 sec and extension at 72 °C for 20 sec, and a final extension step at 72 °C for 3 min. RT-PCR reactions were run on 2% agarose gel and images detected using a Fluochem 8900 imager (Alpha Innotech).

### Protein extraction and Western blot analysis

Cells were washed with PBS (without Ca^2+/^Mg^2+^) and lysed directly in the tissue culture plate on ice or from cell pellets, in ice-cold lysis buffer (25 mM Tris-HCL, pH 7.6, 150 mM NaCl, 1% Triton-X, 1% Na.Deoxycholate) containing protease inhibitor cocktail (Roche Diagnostics). Cell lysates were incubated on ice for 30 min and clarified by centrifugation at 21,000 × *g* at 4 °C for 15 min. Total protein concentrations were determined, using the Bio-Rad Protein Assay (Bio-Rad). The supernatant aliquots containing equal amounts of total protein (40 μg) were diluted 1:2 with Laemmli sample buffer (Bio-Rad) and run on a 10–12% SDS-PAGE at 100 V for 1.5 hr and transferred to a nitrocellulose membrane (Amersham), using a wet transfer apparatus (Bio-Rad) at 80 V for 1 hr at room temperature. Membranes were washed once with TBST (TBS containing 0.05% Tween-20, Sigma) and blocked with 5% skim milk in TBST for 2 hr at room temperature. The membrane was incubated with primary antibodies (see the Supplementary Table 1 for details) overnight at 4 °C with gentle rocking. β-ACTIN (ACTB) (Bio-Rad, 1:5000) was used as a loading control. Membranes were washed three times for 10 min per wash in TBST. All secondary antibodies, anti-rabbit and anti-mouse IgG-HRP conjugates (Bio-Rad, 1:5000), were diluted in 5% skim milk and the membranes were incubated for 1 hr at room temperature. Membranes were washed three times for 10 min per wash in TBST and consequently detected by Clarity chemiluminescence reagent (BioRad). The ECL signal was analyzed by FluorChem 8900 (Alpha Innotech). Rainbow markers (Amersham) were used to determine the protein sizes.

### Flow Cytometry

i-BECs were harvested using Accutase (Invitrogen) for 6 min and fixed using 3% paraformaldehyde (PFA) for 20 min. Cells were blocked with 3% BSA in PBS for 30 min at room temperature and incubated with Human Glut1 Alexa Fluor 700-conjugated Antibody (R&D Systems) at a dilution of 5 µl/1.0 × 10^6^ cells in 800 µl of 1% BSA in PBS and 2 mM EDTA for 1 hr. Cells were washed three times with 1% BSA in PBS and re-suspended in 500 µl 1% BSA in PBS and analyzed on a BD LSRFortessa (BD Biosciences) flow cytometer. Unstained cells were used as a control for autofluorescence and FSC and SSC gating was used to locate cells, exclude doublets and debris and dead cells. Flow cytometry data were analyzed using the FACS Diva software (Becton, Dickinson and Company). Mean fluorescence intensity (MFI) was acquired to measure the shift in fluorescence intensity of GLUT1 during the differentiation process.

### Preparation of transwell inserts and TEER measurements

Transwell inserts (0.9 cm^2^ cell growth area with 1 µm pore size, BD-Falcon, Cat. 353103) with polyethylene terephthalate (PET) membranes were coated with 0.5% gelatin, placed into 12-well companion plates and incubated overnight at 37 °C. The gelatin solution (Sigma) was removed the following day and 5 × 10^5^ i-BECs (at day 10 in EM) were seeded in each transwell insert in 1 ml complete endothelial medium (EM) composed of human endothelial-serum free medium (Life Technologies), supplemented with 1% Platelet-poor plasma derived serum (Alfa-Aesar), 20 ng/ml bFGF (Life Technologies)) and 10 µM Y27362 (ROCK Inhibitor). The inserts were incubated overnight at 37 °C in 5% CO_2_ and the following day, the complete EM medium (without ROCK Inhibitor) was replaced only in the apical cell layer of the transwell insert. For the transport studies, the apical volume is 1 ml and the basolateral volume is 2 ml. Transendothelial electrical resistance (TEER) measurements were carried 16–24 hr following media change using a CellZscope apparatus (Nanoanalytics) with 1 cm diameter electrodes and standard spectrum settings: frequency 1 Hz-100 kHz, points per decade 9 and logarithmic spacing. TEER values for the empty inserts were subtracted from the TEER values for the inserts with cells to obtain final TEER values in Ωcm^2^. Following TEER measurements, cellular viability and BBB integrity were assessed as quality control measures using BBBView, a method where cells on inserts were live stained in Live Cell Imaging Buffer (Invitrogen) containing 2.5 µg/ml of CFDA for 30 min at 37 °C to assess cellular viability. The cells were then placed on ice for 5 min and 5 µg/ml of Cell Mask Orange dye (Invitrogen) was added for 5 min to label the plasma membrane. The cells were washed twice with cold PBS (without Ca^2+/^Mg^2+^) and imaged using a 20 × HMC objective on an Axiovert 200 M Microscope (Zeiss).

### SV-ARBEC BBB model

Immortalized adult rat brain microvascular endothelial cells, SV-ARBECs, were established by SV-40 transfection of primary rat brain microvascular endothelial cells, isolated from 24–30 day old Sprague-Dawley rats, as previously described^49^. The cells were grown in M199 based medium (Wisent) and routinely passaged at a split ratio of 1:20 every week. For transport studies, the SV-ARBEC cells were seeded at 80,000 cells/membrane on rat-tail collagen I (VWR)-coated 0.9 cm^2^ Falcon cell inserts, 1 µm pore size (same as for the iBEC cells), in 1 ml SV-ARBEC feeding medium without phenol red (Invitrogen). The SV-ARBEC BBB model characterization and transport experiments were performed as previously described^49,51^.

### Permeability transport assay

A 2 × input solution of 1 µCi/ml (0.575 µM) of ^14^C -Sucrose (Perkin Elmer), and 3 µCi/ml (0.389 µM) of ^3^H-Cyclosporine A (Perkin Elmer), 3 µCi/ml (3.75 µM) of ^3^H -Diazepam (ARC) and 3 µCi/ml (5.00 µM) ^3^H- l -H-Dopamine (ARC) was prepared in transport buffer (5 mM MgCl_2_ and 10 mM HEPES in HBSS, pH 7.4) and warmed to 37 °C. All radiolabeled compounds were dissolved in ethanol as per manufacturer’s instructions and three blank inserts were used in each experiment. The 12-well transwell inserts (BD-Falcon) containing a confluent monolayer of i-BECs were dipped sequentially for three consecutive washes 5–10 min in wells containing 2 ml pre-warmed HBSS to remove any residual medium. The inserts were then placed into companion plates containing 2 ml of pre-warmed transport buffer, equilibrated to 37 °C in an incubator for 5–10 min and then 500 µl of the media was carefully removed from the top (apical) chamber of each insert and replaced with 500 µl of 2 × input solution. The inserts were incubated at 37 °C with gentle rotation using the 311DS Labnet (Labnet International Inc.) incubator containing orbital shaking platform set at 20 rpms for an hour and 100 µl of transport buffer was collected from the bottom (basal) wells at 15, 30, 45 and 60 min intervals for permeability analysis. Following each collection, 100 µl pre-warmed transport buffer were added back to the bottom wells and the plates were returned to the incubator. The sample collection was also carried out in 3 inserts without cells at 3, 7, 10, 15, 20, 30, 45 and 60 min and clearance slopes were calculated from the linear portion of the curves (0–30 min). Samples were collected in 24-well microbeta sample plates (Perkin Elmer). Duplicate 2 × final concentration input solution was also collected by adding 10 µl of each respective input solution to each well and 90 µl transport buffer. For quantitation, 400 µl high aqueous capacity scintillation fluid was added to each sample well, the plate covered with a microbeta plate seal and contents mixed well by gently shaking the plate until solution was homogeneous and clear. The amount of radioactivity per sample was counted in a scintillation counter (Perkin Elmer), using normalized protocol: ^14^C and ^3^H channel, 2 min per well, disintegration per minute (dpm). The permeability coefficient (Pe) calculations are described in Supplementary Methods.

### Quantification of antibodies in transport assay, CSF and serum samples using multiple reaction monitoring (MRM)

All pure antibodies and proteins from *in vitro* transport samples or body fluids samples were reduced, alkylated and trypsin digested using a previously described protocol^50,51^. For isotopic dilution-based quantification, isotopically heavy versions of the peptides were synthesized from a commercial source (New England Peptide LLC, Gardner, MA) that contained heavy C-terminus K (+8 Da). To develop the SRM assay for proteins, each protein was first analyzed by nanoLC-MS/MS using data-dependent acquisition to identify all ionizable peptides. For each peptide, 3 to 5 of the most intense fragment ions were chosen. An initial MRM assay was developed to monitor these fragments at attomole amounts of the digest (about 100–300 amol). Fragments that showed reproducible intensity ratios at low amounts (i.e., had Pearson r^2^ ≥ 0.95 compared to higher amounts) were considered stable and were chosen for the final MRM assay (see Supplementary Table 3). The apparent permeability coefficient (P_APP_) and apparent CNS exposure (EXP_APP_) values were calculated, as described previously^50,51^.

### Electrophysiological recordings

Coverslips plated with i-Ns were transferred to a recording chamber under a sterile hood and then to an Axiovert 135 inverted microscope (Carl Zeiss) for electrophysiological recordings. The cells were perfused using gravity (~ 2 ml/min) with an extracellular recording solution containing 150 nM NaCl, 4 nM KCl, 1.2 nM CaCl_2_, 1 nM MgCl_2_, 10 nM HEPES (300 mOsmol; pH = 7.4). Whole-cell patch clamp recordings were performed at room temperature using borosilicate pipettes obtained with a Flaming/Brown Micropipette Puller (P-97 Sutter Instruments) from 1.2 mm OD/0.94 mm ID glass capillaries (Warner Instruments). The pipettes were fire polished and had a resistance of 3–4 MΩ when filled with an internal solution containing 115 nM K-gluconate, 10 nM HEPES, 7 nM KCl, 0.05 nM EGTA, 2 nM Na_2_ATP, 2 nM MgATP, 0.5 nM Na_2_GTP (pH 7.3; 290–295 mOsmol/kg). Current-clamp recordings were carried out using Axopatch 200B amplifier (Molecular Devices) and digitalized using Digidata 1322 A (Molecular Devices) connected to a computer equipped with pClamp 10 software (Molecular Devices). Recordings were digitized at 20 kHz and filtered at 10 kHz. Voltage measurements were performed at fixed time intervals (2 sec) with respect to the onset of current pulses, changes in membrane potential were generated by injecting incremental current steps. Series resistance (R_S_) was monitored in response to a 10 mV voltage step and recording with R_S_ greater than 30 MΩ were discarded. Analyses were carried out off-line with the software IGOR (Wavemetrics Wavemetrics) and Clampfit 10 (Molecular Devices).

### Cell uptake assays

i-BECs (300 000 cells/well) were plated on Matrigel coated coverslips in 24 well plates, washed twice with medium and 10 µg/ml acetylated-LDL (Molecular Probes) conjugated with Alexa Fluor 488 (Invitrogen) was added to the wells and incubated for 6 hr at 37 °C. The cells were washed twice with PBS (without Ca^2+/^Mg^2+^), and fixed with 10% formalin in PBS for 10 min. Coverslips were stained with Hoechst 33258 (Sigma) and mounted in DAKO fluorescent mounting medium. For the internalization studies of FC5-Fc and IGF1R-Fc, the i-BECs were grown on Matrigel coated 12 mm round coverslips in 24 well plates in complete EM. Growth medium was removed and 300 µl of cold human endothelial – serum free growth medium (HE-SFM, ThermoFisher) basal medium added to the coverslips and cells cooled to 4 °C by placing the plate on ice for 5 min. FC5-Fc and IGF1R-Fc single domain antibodies, conjugated to a fluorescent dye Alexa Fluor 680 (Al680), were prepared in equimolar (5 µM) concentrations in cold HE-SFM basal medium. The medium was removed from the wells and replaced with 300 µl per coverslip of either Neutralized Al680 or FC5/IGF1R-Fc -Al680 and incubated, on ice in the dark, for 15 min. The cells were washed with 1 ml of cold HE-SFM medium/ well for 1 min and then incubated in 300 µl of cold HE-SFM medium/ well at 37 °C for 60 min. Cells were then washed in cold HE-SFM medium/well and coverslips fixed with 10% Formalin for 10 min at room temperature followed by two washes with PBS. To label the plasma membranes, cells were stained with wheat germ agglutinin-FITC (WGA-FITC) 1:2000 dilution in PBS for 1 min on ice, washed for 5 min with PBS and mounted with Dako Fluorescent Mounting Medium spiked with 2 µg/ml of Hoechst33342 onto the slide. Images were acquired using 63 × oil immersion objective with Zeiss microscope.

### Statistical Analysis

At least two independent differentiations and three technical experiments were performed. Results are given as mean ± standard deviation (SD), or if indicated, as standard error of mean (SEM). Statistical test are indicated in Figure legends and level significance was set at p < 0.05, indicated with asterisk (*). Grading in significance is indicated as follows: *p < 0.05, **p < 0.01, ***p < 0.001.

### Data availability

The datasets generated during and/or analyzed during the current study are available from the corresponding author on reasonable request.

## Electronic supplementary material

Supplementary Information

## References

[1] Arrowsmith J, Miller P (2013). Trial watch: phase II and phase III attrition rates 2011-2012. Nat Rev Drug Discov.

[2] Dawson GR, Dourish CT, Goodwin GM (2011). Special issue on CNS experimental medicine. J Psychopharmacol.

[3] Palmer AM, Alavijeh MS (2012). Translational CNS medicines research. Drug Discov Today.

[4] Gabathuler R (2010). Approaches to transport therapeutic drugs across the blood-brain barrier to treat brain diseases. Neurobiol Dis.

[5] Neuwelt EA (2011). Engaging neuroscience to advance translational research in brain barrier biology. Nat Rev Neurosci.

[6] Zhao Z, Nelson AR, Betsholtz C, Zlokovic BV (2015). Establishment and Dysfunction of the Blood-Brain Barrier. Cell.

[7] Deeken JF, Loscher W (2007). The blood-brain barrier and cancer: transporters, treatment, and Trojan horses. Clin Cancer Res.

[8] Pardridge WM (2003). Blood-brain barrier drug targeting: the future of brain drug development. Mol Interv.

[9] Abbott NJ, Patabendige AA, Dolman DE, Yusof SR, Begley DJ (2010). Structure and function of the blood-brain barrier. Neurobiol Dis.

[10] Ghose AK, Viswanadhan VN, Wendoloski JJ (1999). A knowledge-based approach in designing combinatorial or medicinal chemistry libraries for drug discovery. 1. A qualitative and quantitative characterization of known drug databases. J Comb Chem.

[11] Deli MA, Abraham CS, Kataoka Y, Niwa M (2005). Permeability studies on *in vitro* blood-brain barrier models: physiology, pathology, and pharmacology. Cell Mol Neurobiol.

[12] Helms HC (2016). *In vitro* models of the blood-brain barrier: An overview of commonly used brain endothelial cell culture models and guidelines for their use. J Cereb Blood Flow Metab.

[13] Bernas MJ (2010). Establishment of primary cultures of human brain microvascular endothelial cells to provide an *in vitro* cellular model of the blood-brain barrier. Nat Protoc.

[14] Rubin LL (1991). A cell culture model of the blood-brain barrier. J Cell Biol.

[15] Durieu-Trautmann O (1991). Immortalization of brain capillary endothelial cells with maintenance of structural characteristics of the blood-brain barrier endothelium. In Vitro Cell Dev Biol.

[16] Muruganandam A, Herx LM, Monette R, Durkin JP, Stanimirovic DB (1997). Development of immortalized human cerebromicrovascular endothelial cell line as an *in vitro* model of the human blood-brain barrier. FASEB J.

[17] Stins MF, Badger J, Sik Kim K (2001). Bacterial invasion and transcytosis in transfected human brain microvascular endothelial cells. Microb Pathog.

[18] Weksler BB (2005). Blood-brain barrier-specific properties of a human adult brain endothelial cell line. FASEB J.

[19] Cecchelli R (2007). Modelling of the blood-brain barrier in drug discovery and development. Nat Rev Drug Discov.

[20] Canfield SG (2017). An isogenic blood-brain barrier model comprising brain endothelial cells, astrocytes, and neurons derived from human induced pluripotent stem cells. J Neurochem.

[21] Lippmann ES, Al-Ahmad A, Palecek SP, Shusta EV (2013). Modeling the blood-brain barrier using stem cell sources. Fluids Barriers CNS.

[22] Yamamizu K (2017). *In Vitro* Modeling of Blood-Brain Barrier with Human iPSC-Derived Endothelial Cells, Pericytes, Neurons, and Astrocytes via Notch Signaling. Stem Cell Reports.

[23] Boyer-Di Ponio J (2014). Instruction of circulating endothelial progenitors *in vitro* towards specialized blood-brain barrier and arterial phenotypes. PLoS One.

[24] Cecchelli R (2014). A stable and reproducible human blood-brain barrier model derived from hematopoietic stem cells. PLoS One.

[25] Katt ME, Xu ZS, Gerecht S, Searson PC (2016). Human Brain Microvascular Endothelial Cells Derived from the BC1 iPS Cell Line Exhibit a Blood-Brain Barrier Phenotype. PLoS One.

[26] Lippmann ES (2012). Derivation of blood-brain barrier endothelial cells from human pluripotent stem cells. Nat Biotechnol.

[27] Yu J (2009). Human induced pluripotent stem cells free of vector and transgene sequences. Science.

[28] Drozd AM (2015). Generation of human iPSCs from cells of fibroblastic and epithelial origin by means of the oriP/EBNA-1 episomal reprogramming system. Stem Cell Res Ther.

[29] Galende E (2010). Amniotic fluid cells are more efficiently reprogrammed to pluripotency than adult cells. Cell Reprogram.

[30] Li C (2009). Pluripotency can be rapidly and efficiently induced in human amniotic fluid-derived cells. Hum Mol Genet.

[31] Chan EM (2009). Live cell imaging distinguishes bona fide human iPS cells from partially reprogrammed cells. Nat Biotechnol.

[32] Abujarour R (2013). Optimized surface markers for the prospective isolation of high-quality hiPSCs using flow cytometry selection. Sci Rep.

[33] Needham K, Hyakumura T, Gunewardene N, Dottori M, Nayagam BA (2014). Electrophysiological properties of neurosensory progenitors derived from human embryonic stem cells. Stem Cell Res.

[34] Ludwig TE (2006). Derivation of human embryonic stem cells in defined conditions. Nat Biotechnol.

[35] Waymouth C (1970). Osmolality of mammalian blood and of media for culture of mammalian cells. In Vitro.

[36] Blak, A. A. & Louis, S. A. (Google Patents, 2012).

[37] Hormia M, Lehto VP, Virtanen I (1983). Identification of UEA I-binding surface glycoproteins of cultured human endothelial cells. Cell Biol Int Rep.

[38] Daneman R (2009). Wnt/beta-catenin signaling is required for CNS, but not non-CNS, angiogenesis. Proc Natl Acad Sci USA.

[39] Ferreira Tojais N (2014). Frizzled7 controls vascular permeability through the Wnt-canonical pathway and cross-talk with endothelial cell junction complexes. Cardiovasc Res.

[40] Lee S (2015). Deficiency of endothelium-specific transcription factor Sox17 induces intracranial aneurysm. Circulation.

[41] Lengfeld JE (2017). Endothelial Wnt/beta-catenin signaling reduces immune cell infiltration in multiple sclerosis. Proc Natl Acad Sci USA.

[42] Lippmann ES, Al-Ahmad A, Azarin SM, Palecek SP, Shusta EV (2014). A retinoic acid-enhanced, multicellular human blood-brain barrier model derived from stem cell sources. Sci Rep.

[43] Cohen-Kashi Malina K, Cooper I, Teichberg VI (2009). Closing the gap between the *in-vivo* and *in-vitro* blood-brain barrier tightness. Brain Res.

[44] Perriere N (2007). A functional *in vitro* model of rat blood-brain barrier for molecular analysis of efflux transporters. Brain Res.

[45] Dehouck B (1997). A new function for the LDL receptor: transcytosis of LDL across the blood-brain barrier. J Cell Biol.

[46] Descamps L, Dehouck MP, Torpier G, Cecchelli R (1996). Receptor-mediated transcytosis of transferrin through blood-brain barrier endothelial cells. Am J Physiol.

[47] Duffy KR, Pardridge WM (1987). Blood-brain barrier transcytosis of insulin in developing rabbits. Brain Res.

[48] Golden PL, Maccagnan TJ, Pardridge WM (1997). Human blood-brain barrier leptin receptor. Binding and endocytosis in isolated human brain microvessels. J Clin Invest.

[49] Garberg P (2005). *In vitro* models for the blood-brain barrier. Toxicol In Vitro.

[50] Farrington GK (2014). A novel platform for engineering blood-brain barrier-crossing bispecific biologics. FASEB J.

[51] Haqqani AS (2012). Multiplexed evaluation of serum and CSF pharmacokinetics of brain-targeting single-domain antibodies using a NanoLC-SRM-ILIS method. Mol Pharm.

[52] Stanimirovic DB, Bani-Yaghoub M, Perkins M, Haqqani AS (2015). Blood-brain barrier models: *in vitro* to *in vivo* translation in preclinical development of CNS-targeting biotherapeutics. Expert Opin Drug Discov.

[53] Forster C (2008). Differential effects of hydrocortisone and TNFalpha on tight junction proteins in an *in vitro* model of the human blood-brain barrier. J Physiol.

[54] Appelt-Menzel A (2017). Establishment of a Human Blood-Brain Barrier Co-culture Model Mimicking the Neurovascular Unit Using Induced Pluri- and MultipotentStem Cells. Stem Cell Reports.

[55] Beauchesne, E., Desjardins, P., Butterworth, R. F. & Hazell, A. S. Up-regulation of caveolin-1 and blood-brain barrier breakdown are attenuated by N-acetylcysteine in thiamine deficiency. *Neurochem Int* **57**, 830–837 (2010). 10.1016/j.neuint.2010.08.022 20816907

[56] Nag S, Venugopalan R, Stewart DJ (2007). Increased caveolin-1 expression precedes decreased expression of occludin and claudin-5 during blood-brain barrier breakdown. Acta Neuropathol.

[57] Drouin-Ouellet J (2015). Cerebrovascular and blood-brain barrier impairments in Huntington’s disease: Potential implications for its pathophysiology. Ann Neurol.

[58] Andreone BJ (2017). Blood-Brain Barrier Permeability Is Regulated by Lipid Transport-Dependent Suppression of Caveolae-Mediated Transcytosis. Neuron.

[59] Agarwal S (2012). Quantitative proteomics of transporter expression in brain capillary endothelial cells isolated from P-glycoprotein (P-gp), breast cancer resistance protein (Bcrp), and P-gp/Bcrp knockout mice. Drug Metab Dispos.

[60] Ito K (2011). Quantitative membrane protein expression at the blood-brain barrier of adult and younger cynomolgus monkeys. J Pharm Sci.

[61] Kamiie J (2008). Quantitative atlas of membrane transporter proteins: development and application of a highly sensitive simultaneous LC/MS/MS method combined with novel in-silico peptide selection criteria. Pharm Res.

[62] Uchida Y (2011). Quantitative targeted absolute proteomics of human blood-brain barrier transporters and receptors. J Neurochem.

[63] Hoshi Y (2013). Quantitative atlas of blood-brain barrier transporters, receptors, and tight junction proteins in rats and common marmoset. J Pharm Sci.

[64] Shawahna R (2011). Transcriptomic and quantitative proteomic analysis of transporters and drug metabolizing enzymes in freshly isolated human brain microvessels. Mol Pharm.

[65] Webster CI (2016). Brain penetration, target engagement, and disposition of the blood-brain barrier-crossing bispecific antibody antagonist of metabotropic glutamate receptor type 1. FASEB J.

[66] Lim RG (2017). Huntington’s Disease iPSC-Derived Brain Microvascular Endothelial Cells Reveal WNT-Mediated Angiogenic and Blood-Brain Barrier Deficits. Cell Rep.

[67] Patel R, Page S, Al-Ahmad AJ (2017). Isogenic blood-brain barrier models based on patient-derived stem cells display inter-individual differences in cell maturation and functionality. J Neurochem.

[68] Sikorska M (2008). Epigenetic modifications of SOX2 enhancers, SRR1 and SRR2, correlate with *in vitro* neural differentiation. J Neurosci Res.

