# Supplementary material for: A novel human induced pluripotent stem cell blood-brain barrier model: Applicability to study antibody-triggered receptor-mediated transcytosis
Source: Sci Rep. 2018 Jan 30;8:1873. doi: 10.1038/s41598-018-19522-8 (PMC5789839; doi:10.1038/s41598-018-19522-8)
Supplement: Supplementary file 1 — Supplementary Information [file 41598_2018_19522_MOESM1_ESM.pdf]

A novel human induced pluripotent stem cell blood-brain barrier model: Applicability to study antibody-triggered receptor-mediated transcytosis

Maria Ribecco-Lutkiewicz, Caroline Sodja, Julie Haukenfrers, Arsalan S Haqqani, Dao Ly, Peter Zachar, Ewa Baumann, Marguerite Ball, Jez Huang, Marina Rukhlova, Marzia Martina, Qing Liu, Danica Stanimirovic, Anna Jezierski*, Mahmud Bani-Yaghoub

Human Health Therapeutics Portfolio, National Research Council of Canada, Ottawa, ON, K1A 0R6, Canada

*Corresponding author:

Dr. Anna Jezierski

National Research Council Canada,

1200 Montreal Rd., Bldg. M-54,

Ottawa, ON, K1A 0R6, Canada

Tel: 613-990-1999

Email: anna.jezierski@nrc.ca

Supplementary Methods

DNA isolation and methylation assay

DNA was isolated from 1.0 x10^6 AF cells and iPSC cells using the Quick gDNA Miniprep (Zymo Research), according to the manufacturer's protocol. The EZ DNA Methylation gold Kit (Zymo Research) was used to modify unmethylated cytosine residues to thymidine. Briefly, the conversion of approximately 300 ng of genomic DNA was carried out in a thermal cycler at 98°C for 10 min, 64°C for 2.5 hr and then stored at 4°C for up to 20 hr. The reaction was then followed by a clean-up, desulfonation and the bisulfite-modified DNA was eluted and immediately used for PCR amplification. Briefly, 2 µl of bisulfite converted DNA was added to a solution containing 1X MSB PCR buffer (16.6 mM ammonium sulfate, 67 mM Tris, pH 8.8, 6.7 mM $MgCl_2$, 10 mM 2-mercaptoethanol), 2.5 µl of DMSO, dNTPs (1.25 mM each), primers (1 µM each) and 2.5 U Platinum Taq DNA Polymerase High Fidelity in a final volume of 50 µl (Life Technologies). The primers were specific for bisulfite-converted DNA only (Supplementary Table 2). The PCR reactions were carried out in a Biorad DNA Engine Dyad thermal cycler using the following protocol: 1 cycle of denaturation at 95 °C for 3 min, 35 cycles of amplification at 95 °C for 1 min, 55–58 °C for 1 min, 72 °C for 1 min and 1 cycle of extension at 72 °C for 10 min. PCR products were loaded onto a 2% agarose gel and the amplicon band was excised and purified using the Geneclean Spin Kit (MP Biomedicals), as per the manufacturer's instructions. Zero Blunt TOPO PCR Cloning Kit (Life Technologies) was used for cloning the PCR product obtained and clones were PCR screened with M13 primers (Supplementary Table 2) for positive transformants. At least 10 positive clones were cultured overnight in 2 ml of LB medium containing 50 µg/ml Kanamycin (Sigma) in a 37°C shaking incubator at 200 rpm. The next day, plasmid DNA was isolated using the Genelute Plasmid

Mini-Prep Kit (Sigma) and sent for sequencing on an ABI 377 sequencer. The sequence was analyzed using Sequencher version 5.2.3 software (Genecode). The sequence of the primers used for Bisulfite (BS) PCR are from[69] (Supplementary Table 2) and M13F and M13R are included in the Zero Blunt TOPO PCR Cloning Kit.

Microarray printing, cDNA synthesis and hybridization

A BBB specific oligonucleotide set of 434 oligos (Eurofins) was printed, in house, onto Nexterion E epoxysilane coated slides (Applied Microarrays) with a Nano-plotter NP2.1(GeSiM) at 50% humidity and a temperature of 8°C. The slides were transferred to a 42°C oven and incubated at 80% humidity overnight then stored in a desiccator (Bel-Art) until ready for use. cDNA was prepared according to the 3DNA array detection 900 kit recommendations (Genisphere), using 1µg of total RNA from each sample and Superscript II Reverse Transcriptase (Life Technologies). The slides were washed and blocked according to the manufacturer's recommendations, spun dry at 200 x g for 5 min and placed in a SlideBooster Microarray Hybridisation Station SB401 (Beckman Coulter). The cDNA hybridization mix was pipetted on top of the slides and then covered with Lifter Slips (Thermo Scientific) and the hybridization reaction was carried out at 42 °C overnight. The slides were then washed in 2X SSC, 0.2% SDS; 2X SSC and 0.2XSSC each for 15 min at room temperature on a shaker then spun dry. The 3DNA capture reagents were prepared, as per manufacturer's protocol, then pipetted onto the slides in the SlideBooster Hybridization Station, as previously described and incubated at 42 °C for 4 hr. The slides were then washed as described above and scanned immediately with the GenePix scanner 4200A (Molecular Devices), as per the manufacturer's

protocol and the data was analyzed using the GenePix Pro 6.1 software (Molecular Devices). Individual gene intensity values for the genes assessed are listed in Supplementary Table 4.

Permeability coefficient (Pe) calculations

Permeability coefficient (Pe) is a parameter that is determined in an *in vitro* or *ex vivo* transport assay system that represents the permeability of a compound across the cell monolayer only. For this calculation, the barrier effect of the cell membrane/insert alone (empty inserts) is subtracted from the overall barrier effect of the cells plus membrane (inserts with cells). Permeability coefficients are calculated from each insert separately and from the pooled data of three inserts in each experiment. Clearance volumes (µl) are calculated as previously described[49]:

clearance (µl) = ([C]A x VA)/[C]D, where

[C]A is the input compound concentration in the accepting chamber (corrected for the sampling aliquots removed in previous time points), VA is the volume in the accepting chamber and [C]D is the initial concentration of input compound in the donor chamber. The slopes of the clearance curves (clearance vs time) for empty membrane and membrane with cell monolayers, representing permeability x surface area product (PS; µl/min), are calculated using linear regression analysis. The PS value for endothelial monolayer (PSe) is calculated from:

1/PSe = 1/PSc+m - 1/PSm, where

PSc+m is the slope of the clearance curve for cell monolayer and membrane together, and PSm is the slope of the clearance curve of membrane alone (ie. no cells). The permeability coefficient (Pe; cm/min) for the endothelial cell monolayer is calculated by dividing PSe by surface area of the membrane (0.9 cm^2 for our inserts).

Supplementary Tables

Table 1. Antibodies/Reagents for immunofluorescence staining

Antibody/Reagent	Supplier	Source	Dilution	Secondary Antibody
OCT4	Santa Cruz sc-5279	Mouse Monoclonal	1:100	Rhodamine conjugated rabbit anti-mouse IgG
SOX2	In house	Rabbit Polyclonal	1:5000	Rhodamine conjugated goat anti rabbit IgG
NANOG	R&D Systems AF1997	Goat Polyclonal	1:100	ALEXA568 conjugated rabbit anti goat IgG
TRA-1-81	Stemgent 09-0069	Mouse Monoclonal	1:100	Directly conjugated to DyLight 488
KLF4	Abcam Ab151733	Rabbit Monoclonal	1:500	Rhodamine conjugated goat anti rabbit IgG
SSEA3	R&D Systems MC-631	Rat Polyclonal	1:20	ALEXA488 goat conjugated anti rat IgG
SSEA4	R&D Systems MC-813-70	Mouse Monoclonal	1:20	Rhodamine conjugated rabbit anti mouse IgG
CD30	BD Biosciences 562876	Mouse monoclonal	1:20	Antibody directly conjugated to BV421
Nestin	Millipore ABD69	Rabbit Polyclonal	1:200	Rhodamine conjugated goat anti rabbit IgG
MAP2	Sigma M1406	Mouse Monoclonal	1:100	Rhodamine conjugated rabbit anti mouse IgG
NCAM	Millipore Ab 5032	Rabbit Polyclonal	1:100	ALEXA488 conjugated goat anti rabbit IgG
Blll-Tubulin	Millipore MAB1637	Mouse Monoclonal	1:200	Rhodamine conjugated rabbit anti mouse IgG
VGLUT1	Millipore MAB5502	Mouse Monoclonal	1:100	Rhodamine conjugated rabbit anti mouse IgG
VGLUT2	Millipore MAB5504	Mouse Monoclonal	1:100	Rhodamine conjugated rabbit anti mouse IgG
GFAP	DAKO Z0334	Rabbit Polyclonal	1:500	ALEXA488 conjugated goat anti rabbit IgG
NeuN	Abcam Ab104225	Rabbit Polyclonal	1:500	Rhodamine conjugated goat anti rabbit IgG
Synaptophysin	Sigma SVP-38	Mouse Monoclonal	1:200	Rhodamine conjugated rabbit anti mouse IgG
Synaptotagmin	Stressgen SYA-130	Mouse Monoclonal	1:100	Rhodamine conjugated rabbit anti mouse IgG
SOX17	R&D Systems AF1924	Goat Polyclonal	1:100	ALEXA568 conjugated rabbit anti goat IgG
PAX6	Covance	Rabbit	1:50	Rhodamine conjugated goat

	PRB-278P	Polyclonal		anti rabbit IgG
ZO-1	Abcam Ab59720	Rabbit Polyclonal	1:100	Rhodamine conjugated goat anti rabbit IgG
Claudin-5	Abcam Ab53765	Rabbit Polyclonal	1:100	Rhodamine conjugated goat anti rabbit IgG
VWF	DAKO A0082	Rabbit Polyclonal	1:200	Rhodamine conjugated goat anti rabbit IgG
CD31	Abcam Ab28364	Rabbit Polyclonal	1:20	Rhodamine conjugated goat anti rabbit IgG
Occludin	Abcam Ab31721	Rabbit Polyclonal	1:100	Rhodamine conjugated goat anti rabbit IgG
GLUT1 (immune)	Abcam Ab40084	Mouse Monoclonal	1:100	Rhodamine conjugated rabbit anti mouse IgG
GLUT1 (Flow)	R&D Systems FAB1418N	Human	5µl/10^6 cells	Human Glut1 Alexa Fluor 700-conjugated Antibody
TfR	Abcam Ab84036	Rabbit Polyclonal	1:100	Rhodamine conjugated goat anti rabbit IgG
IGF1R	Cell Signaling 3027S	Rabbit Polyclonal	1:100	Rhodamine conjugated goat anti rabbit IgG
LRP1	Abcam Ab92544	Rabbit Polyclonal	1:100	Rhodamine conjugated goat anti rabbit IgG
ULEX	Vector Labs FL-1061		1:500	Directly conjugated to FITC
Wheat Germ Agglutinin	Vector Labs FL-1021		1:2000	Directly conjugated to FITC
DAKO Protein Block Serum Free	Agilent X0909			
DAKO Antibody Diluent	Agilent S0809			
DAKO Fluorescence Mounting Medium	Agilent S30238			

Table 2. Primers used for RT-PCR

Gene	Forward Sequence	Reverse Sequence
**Primers for BBB Characterization**		
GAPDH	GAGAAGGCTGGGGCTCATTTG	GGTGCTAAGCAGTTGGTGGT

SLC2A1	ACGCTCTGATCCCTCTCAGT	GCAGTACACACCGATGATGAAG
ABCB1	TGAATCTGGAGGAAGACATGAC	CCAGGCACCAAAATGAAACC
LEF1	CAGATGTCAACTCCAAACAAGG	GATGGGATATACAGGCTGACC
STRA6	TTTGGAATCGTGCTCTCCG	AAGGTGAGTAAGCAGGACAAG
FZD4	TACCTCACAAAACCCCCATCC	GGCTGTATAAGCCAGCATCAT
FZD6	TCGTCAGTACCATATCCCATG	CCCATTCTGTGCATGTCTTTT
FZD7	GATGATAACGGCGATGTGA	AACAAAGCAGCCACCGCAGAC
FST	GTTCATGGAGGACCGCAGTG	TCTTCTTGTTCATTCGGCATT
APCDD1	GGAGTCACAGTGCCATCACAT	CCTGACCTTACTTCACAGCCT
PECAM1	GCAAAATGGGAAGAACCTGA	CACTCCTTCCACCAACACCT
CDH5	CGCAATAGACAAGGACATAACAC	GGTCAAACTGCCCATACTTG
VWF	CCCGAAAGGCCAGGTGTA	AGCAAGCTTCCGGGGACT
LDLR	GCCATTGTCGTCTTTATGTC	AAACACATACCCATCAACGA
SLC7A5	TTAAAGTAGATCACCTCCTCGA	GGATGAGATTCGTACCAGAG
SLC16A1	GGTGTTTCTTAGTAGTTATGGG	TCTTATTGGCTTTGTGTTGG
SLC15A2	TTTTTGTGCTTCAGCCGGAC	GAACTGTGCCACAACAAGCA
INSR	TGTTCATCCTCTGATTCTCTG	GCTTAGATGTTCCCAAAGTC
LEPR	GGAAATCACACGAAATTCAC	GCACGATATTTACTTTGCTC
BCAM	GCTTTCCTTACCTCTAAACAG	GAAGGTGATAGAACTGAGCG
SLC38A5	TGTCAGTGTTCAACCTCAG	GTGGATGGAGTAGGACGA
SLC28A1	ACCTCGTTGATTGCAGCCTC	GTGGTTGTAAAACCGACAGCAG
SLC29A3	ATTGATTCCCACACACCCCC	GAGCCCAGTGTTAAGCCCAA
SLC1A1	GTTATTCTAGGTATTGTGCTGG	CTGATGAGATCTAACATGGC
ABCG2	TCAGGTCTGTTGGTCAATCTC	GTTTCCTGTTGCATTGAGTCC

ABCC1	AATAGAAGTGTTGGGCTGAG	CGAGACACCTTAAAGAACAG
ABCC2	TGCTTCCTGGGGATAATCAG	CACGGATAACTGGCAAACCT
ABCC4	AATCTACAACTCGGAGTCCA	CAAGCCTCTGAATGTAAATCC
ABCC5	TCACTACATTAAGACTCTGTCC	GGATACTTTCTTTAGGACGAGAG
ABCC3	GCAGCACCTGGGAAAAATGC	TGTCAGGGTAGAGTCCAATGAG
AGER	GTAGATTCTGCCTCTGAACTC	CTTCACAGATACTCCCTTCTC
SLC21A14	TGGTTGTCAAACCTCCAACA	AGCTGTGGCTTAATGCACCT
PLVAP	CAATGCAGAGATCAATTCAAGG	ACGCTTTCCTTATCCTTAGTG
TFRC	GCACAGCTCTCCTATTGAAAC	GGTATCCCTCTAGCCATTCAG
LRP1	GACTACATTGAATTTGCCAGCC	TCTTGTGGGCTCGGTTAATG
WNT7A	CGGGAGATCAAGCAGAATG	CGTGGCACTTACATTCCAG
WNT7B	GCTTCGTCAAGTGCAACA	GGAGTGGATGTGCAAAATG
IGF1R	ACAGAGAACCCCAAGACTGAGG	TGATGTTGTAGGTGTCTGCGGC
Tmem30a iso1	GGGCACCGCGAAGA	ATTCAAAGAGTATCGGCCAGCT
Tmem30a iso 2	AGATCGAGGGCAACGTGTTTAT	ATTCAAAGAGTATCGGCCAGCT
CLDN3	CCCACGCGAGAAGAAGTACA	CAAGTATTGGCGGTCACCCA
CLDN5	ATGGGGTCCGCAGCGTTGGAGATCCT	GACGTAGTTCTTCTTGTCGT
ZO-1	AGCTCCACAGGCTTCAGGAAC	CTGTGAGTCCTTCAGCTGTGG
ZO-2	CCGGAGGCAGAGACAACCC	AACTTCTGCCATCAAACTCG
ZO-3	CTCTACGCACAAGCCCAGAA	CCCCAGTCATAGCCGTCTTC
Occludin	AGTACATGGCTGCTGCTGATG	AATTGGAGTGTTCAGCCCAGT

***** All primers are from[26], with the exception of GAPDH, PECAM1, ABCC2, SLC21A14, and Tmem30A isoform1 and isoform 2 which were designed in-house.

Primers for exogenous episomal gene expression (primers from[27])

exoNanog:	CAGAAGGCCTCAGCACCTAC	AGGAACTGCTTCCTTCACGA
exo-c-MYC	TCAAGAGGCGAACACACAAC	AGGAACTGCTTCCTTCACGA

Table 3. Peptide signatures used for SRM detection of various proteins used in the study.

Protein	Peptide Sequence	Charge	m/z of peptide	Peptide fragment signatures (m/z) used for quantification	LOQ (pM) (c)
IGF1R5	TIDNYAMAWSRq	2	664.27	1113.52, 721.41, 448.29, 519.33	1
	LEESGGGLVQAGGSLR	2	765.35	688.42, 787.42, 560.25, 489.33	1
OX26	CPAPNLLGGPSVFIFPPK	2	956.01	1145.46, 1582.65, 1258.53, 341.17	5
	TTAPSVYPLAPVCGDTTGSSVTLGCLVK	2	1425.87	1850.67, 1290.06, 1147.95, 1921.67	5
FC5	ITWGGDNTFYSNSVK	2	844.92	534.48, 729.47, 737.89, 1288.44	25
	ITWGGDNTFYSNSVK(b)	2	848.92	542.48, 733.48, 741.87, 1296.44	25
A20.1	TFSMDPMAWFR	2	694.77	582.22, 807.4, 922.42, 1140.5	33
	TTYYADSVK	2	524.23	203.1, 682.34, 845.4	66
	EFVAAGSSTGR	2	541.29	500.12, 564.32, 635.28, 706.44	50
Human Fc	TTPPVLDSDGSFFLYSK	2	937.28	836.8, 1150.24, 1265.44, 1378.72, 1477.44	8
	TTPPVLDSDGSFFLYSK(b)	2	941.28	840.5, 1158.25, 1273.44, 1386.74, 1485.44	8

Albumin	DNCFATEGPNLVAR	2	782.32	246.16, 346.28, 1174.48	-
	APQVSTPTLVEAAR	2	720.84	703.0, 856.9, 957.9, 1044.8, 1143.7	-

(a) Limits of quantitation (LOQ) is shown in picomoles per litres (pM). LOQ of each peptide was determined from a dilution series (50-2000 attomole) of each protein digest or ILIS peptide analyzed by nanoLC-SRM.
(b) Heavy peptide (isotopically labeled internal standards ILIS)

Table 4. Individual gene intensity values from microarray analysis comparing i-BEC and HBMECs

Gene Name	i-BEC	HBMEC
A2M	8349	8656
ABCA1 (ABC1)	9530	9663
ABCA10	14903	13215
ABCA12 (1)	10454	5029
ABCA12 (2)	2662	2135
ABCA13	44131	86095
ABCA2	52539	38978
ABCA3	57226	49163
ABCA4	34184	34028
ABCA5	50489	45207
ABCA6	2462	2509
ABCA7	54644	56807
ABCA8	40660	23961
ABCA9	55967	44472
ABCB1/MDR-1 PGP	7314	9312
ABCB10	13570	22027
ABCB11	44080	33638
ABCB4	13294	12940
ABCB5	2712	1914
ABCB6	9084	5963
ABCB8	52513	47226
ABCB9 (1)	24700	24818
ABCB9 (2)	52146	51836
ABCC1	40756	33288
ABCC10	6688	7384
ABCC11	28954	29489
ABCC12	18325	18852
ABCC13 (1)	4158	3497

ABCC13 (2)	5568	7748
ABCC2	4261	2648
ABCC3	4731	3248
ABCC5	994	1005
ABCC8	7183	7075
ABCC9	26845	23902
ABCD3	12023	10860
ABCD4 (ALD)	1461	776
ABCF1	10090	11284
ABCF2	41607	34890
ABCF3	15356	15438
ABCF3 (GCN20)	16163	16842
ABCG1	21956	24272
ABCG2	2202	2449
ABCG4	6949	7420
ABCG5	50517	45793
ABCG8	32639	31283
ACCN4	23556	27439
ACTB	49279	46585
AGER	28799	28670
AKAP12 (gravin)	16582	15402
ALPI	49943	42325
ALPL	24631	23510
ALPP	42073	45317
ANGPT1 (V1)	16698	19538
ANGPT1 (V2)	1740	1268
ANO4 (TMEM16D)	8477	9396
ANO6 (TMEM16F)	19618	20717
ANO9 (TMEM16J)	48132	36781
AP2A2	18375	22423
APOB	46766	40121
APOE	65535	66134
APOL1	14228	11133
AQP10	26825	26790
AQP2	19643	19711
ARL6IP5	23101	22370
ATF1	51461	51131
ATP11C	1090	911
ATP13A4	4143	3394

ATP1A1	6028	4531
ATP1A2	5278	3967
ATP1A3	42796	29637
ATP1B1	14969	8599
ATP1B3	8253	8375
ATP2A2	59474	52154
ATP2B1	3574	1674
ATP2B3	47767	66859
ATP6V0D1	19891	34314
ATP6V1B1	16443	14581
ATP7A	3365	5535
ATP8B1	54593	51961
ATP8B2	4510	4618
ATP8B4	10587	10919
BCAP31	51496	46937
BEST4 (VMD2L2)	45071	33023
BPIFA2	56472	50962
BSG	22267	16289
CACNA1A	18013	15915
CACNA1C	2665	3049
CACNA2D1	21178	21970
CACNB2	1273	708
CAV1	8700	53398
CD36	58421	60717
CDH12	3621	4230
CDH5	7484	21230
CFL1	32593	33115
CFTR	52492	50457
CFTR/MRP (ABCC6)	27878	28768
CGN	9942	7061
CHRNA5	7059	7806
CKB	51319	49145
CLCA1	41831	39704
CLCN1	50484	23737
CLCN3	33588	41005
CLDN1	13616	12101
CLDN3	54495	40107
CLDN5	41333	40447
CLIC1	44041	46469

CNGB1	43582	36100
CNTNAP2	2722	1720
CNTNAP4	10591	11438
CNTNAP5	15830	17394
COL4A1	4341	9328
COL4A2	31035	32635
COL4A4	19788	19627
COLEC12 (SRCL)	7787	7986
CPE	33824	27516
CPNE3	2368	1627
CPNE7	57657	52056
CST3	15258	17664
CTNNB1	31476	28060
CTNND1	18076	17082
CTTN	15694	17734
CUBN	51782	39821
CYB5-M	37041	39018
DENND5A (RAB6IP1)	19044	32863
DENND5B	4138	2777
EEA1	1872	1255
EFEMP1	45728	37001
ENG	49003	40614
ENT3 (SLC29A3)	20162	20370
ENT4 (SLC29A4)	48729	40586
EPAS1	12995	7965
ESAM	3831	68266
F11R	61507	59450
F5	30604	28991
FGF19	2672	4340
FGG	64513	51312
FLT1	18181	21447
FN1	9579	5636
GABRA2	7817	9399
GAPDH	57481	61768
GFAP	11962	12914
GGA1	34656	27739
GGTL4	56549	63807
GPRC6A G	6685	6887
GRIK3	16187	16821

GRIN1	43967	41953
GRIN2A	13336	12641
HBA1	58004	51394
HHIPL2	17754	17650
HIF1A	6802	5858
HIST2H2AA4	59803	73709
HPRT1	12133	12829
ICAM-1(CD54)	1664	482
IGF1R	44654	34599
IGF2 (somatomedin A),	38459	35899
IGF2R	25984	28341
IGFBP1	21890	20941
IGFBP2	57082	51311
IGFBP3	14705	4479
IGFBP4	58998	58550
IGFBP5	9629	738
IGFBP6	60427	57553
IKBKE	24299	23652
IL1B	39886	34257
INSR	3478	3694
ITPR2	16101	17537
ITPR3	24649	26033
JAM-B	51438	43400
JAM-C	4517	13210
JUP (CTNNG)	45237	68362
KCNA5	27812	23553
KCNA7	10141	9202
KCNH1	10196	8794
KCNH5	2993	2791
KCNK10	2837	2849
KCNMB2	3060	3677
KCNQ2	5827	5048
KCTD16	2972	2597
KDR	660	1050
LAMA1	17644	19409
LAMA3	7551	5962
LAMA4	35607	38046
LAMB1	7886	19147
LAMB2	45942	35848

LAT1-3TM	8301	7482
LCN1	49168	45325
LRP1	54000	51824
LRP10	16495	18040
LRP11	1893	2205
LRP2	11975	11255
LRP3	55596	46933
LRP4	34971	31935
LRP5	34251	31032
LRP6	11448	14097
LRP-8	5398	5691
LU	38674	37475
M6PR	33551	28995
MACF1	3126	4547
MAGT1	48911	48472
MAOB	30511	23760
MAP1A	37177	32776
MAP1B	36409	33021
MARCO	40996	29262
MB	5571	4998
MBP	30470	29686
MCOLN1	60720	60256
MCP	6928	7630
MCT1 (SLC16A1)	52128	52274
MCT2 (SLC16A7)	7934	14385
MCT3 (SLC16A8)	7400	7687
MF12	6879	6608
MFSD2A	14148	14344
MLLT4, AF6	3845	5337
MMP12	762	687
MMP13	6656	6555
MMP14	28698	27698
MMP2	35153	31792
MMP3	20850	24951
MMP7	26680	38585
MMP8	1900	1950
MMP9	13650	4211
MPZL1	28855	27072
MPZL2 (EVA1)	8270	9390

MRP4 (ABCC4)	4189	3650
MSR1	3814	2957
MVP	65535	58033
MYO5A	6013	8092
NFKB1	4995	4774
NFKBIA	50062	47958
NRP1	6114	8408
NRP2	3428	2046
NRXN1	4471	3260
NRXN2	24929	23882
OATP-H (SLC21A12)	11462	8200
OATPRP3 (SLCO3A1)	13791	10884
OCLN	19013	15837
OSTalpha	2856	2567
OSTbeta	27556	29373
P2RX4	48990	40718
PARD3	21437	23793
PECAM-1 (CD31)	30137	28999
PKD1	61314	59302
PLAT	50499	47558
PLP2	17625	17159
PLSCR1	1705	919
PLSCR3	14784	8354
PLSCR4	2449	7571
PODXL	9563	5931
PRAF2	24458	23804
PRG1	3815	31126
PROCR	43200	45606
PROM1	3584	2487
PROM2	5964	4671
RAB3D	10313	10518
RAB5A	6680	6635
RAMP2	64448	52821
REEP2	61314	65506
REEP5	51117	47451
REEP6	54075	55812
RPH3A	46697	42643
RYR2	18118	19906
S100A6	56696	43557

S100B	22150	21931
SCARA3	9230	8643
SCARB1	13033	11962
SCARB2	14309	14091
SCN10A	10708	7356
SCN3A	633	201
SCN4B	11438	11522
SCN7A	2380	1470
SCN8A	21492	20502
SEC61A1	19366	21265
SEC61A2	15697	16226
SELE	21217	22202
SHROOM3	14267	15902
SLC10A6 (SOAT)	28720	26407
SLC11A2	23056	26645
SLC12A2	38273	27758
SLC12A7	15069	19119
SLC15A1	25513	25614
SLC15A2	27674	26079
SLC16A1	20897	23112
SLC16A10	33524	31495
SLC16A11	63866	69500
SLC16A2	21092	19142
SLC16A3	63464	71174
SLC16A4	58575	53079
SLC16A5	15305	14516
SLC16A6	13211	12132
SLC17A1	5974	7271
SLC17A2	21237	28460
SLC17A3	31366	21281
SLC17A4	21952	22289
SLC17A6	2943	2779
SLC17A7	53135	42474
SLC17A8	12601	11584
SLC18A1	28266	27663
SLC18A2	3800	2954
SLC1A1	3417	4234
SLC1A2	8122	8299
SLC1A3	1470	807

SLC1A4	5421	6155
SLC1A5, ASCT2	52242	40316
SLC1A6	10867	9444
SLC1A7	28679	31107
SLC21A14	5398	6092
SLC21A8	12667	13155
SLC22A1 (hOCT1)	8147	8200
SLC22A10	46954	34361
SLC22A11	45720	37489
SLC22A12	65535	57502
SLC22A13	38639	42435
SLC22A14	31100	28043
SLC22A15	13394	14138
SLC22A17	41874	35278
SLC22A18	28634	15599
SLC22A1LS	33999	29889
SLC22A2	14470	5658
SLC22A3	50791	46681
SLC22A4	46170	42030
SLC22A5	12986	13746
SLC22A7	60221	60313
SLC22A8, hOAT3	3826	4173
SLC22A9	38908	33149
SLC26A5 (Prestin)	36201	28836
SLC26A8	6234	5026
SLC28A1 (CNT1)	32726.61	25783.06
SLC28A3	17326	23214
SLC29A1	53889	48259
SLC29A2	32836	32522
SLC2A1	15563	9324
SLC2A10	22602	27322
SLC2A11	40742	39209
SLC2A12	9990	17202
SLC2A13	21428	28551
SLC2A14	9295	1641
SLC2A4	38926	33246
SLC2A5	17034	16397
SLC2A6	27650	27769
SLC2A8	14762	14033

SLC2A9	50596	46914
SLC30A1	36416	29977
SLC30A8	38001	31557
SLC35B2	47730	44539
SLC35B4	19859	14863
SLC37A4	11594	11580
SLC38A1	11893	6531
SLC38A2 (ATA2)	3580	1956
SLC38A4	3086	2561
SLC3A2	56368	55228
SLC41A2	14841	15061
SLC44A3	1825	1232
SLC5A10	47124	44198
SLC5A7	1756	783
SLC6A13	34552	33501
SLC6A6	14331	15699
SLC7A1	2015	1163
SLC7A2	31471	30497
SLC7A3	33521	27056
SLC7A4	64650	57200
SLC7A5	37829	29465
SLC7A6	4052	4766
SLC7A7	60169	44632
SLC7A8	26810	28287
SLC7A9	12353	13283
SLC9A5	59421	44703
SLC9B1	19190	17865
SLCO1A2	25135	27278
SLCO1B1	42509	38146
SLCO2A1	1759	713
SLCO2B1	33733	29640
SLCO3A1	17881	16671
SLCO4A1	4940	5426
SLCO5A1	49669	35674
SLCO6A1 (OATP-I)	8141	7993
SMARCA4	48114	42079
SORCS1	4104	4132
SORCS3	9438	9766
SORL1	7224	5918

SOX17	8710	10257
SPARC (osteonectin)	15130	35268
STAB1	49811	37477
SUSD1	4220	4759
SVOPL	14766	15535
SXR (NR1I2)	27231	24001
SYPL1	12561	14156
TAP1	55286	53246
TAP2	7244	9679
TAPBP	13112	13264
TFR2	4724	4871
TFRC	5689	4533
THBS2	4530	3659
TIMP1	56238	56648
TIMP2	8798	11343
TIMP3	9057	6130
TIMP4	37797	42086
TJP1	16579	18904
TJP2	9399	9055
TJP3	45813	38917
TMEM30A (CDC50)	10619	12022
TNF-a	4285	4681
TOM1L1	8194	7508
TRPC3	12845	19686
TRPC4	14691	16219
TRPM2	13091	11956
TRPM3	47014	46409
TRPM7	35931	32061
TRPV5	45470	44971
TUBA3	29036	16571
VCAM1	42580	29929
VEGF-A	6908	6131
VIM	17214	24242
VLDLR	7339	6759
VWF	42099	29804
WNK1	12775	11552
YBX3 (CSDA)	16955	22804
ZER1	53725	32627

Microarray experiments were performed on triplicate cell samples, where HBMEC was used as a control during data analysis. For the i-BEC cells, 3 biological replicates were used in the analysis from 3 independent differentiation cultures. Dye-swap was done during hybridization, which resulted in 6 direct comparisons between samples. Signal intensity of each genes was normalized against the average intensity of 10 housekeeping genes that were included on the microarray slides (GUSB, HMBS, PES1, PGK1, POP4, PPIA, PPIB, RPL30, RPL37A and RPLP2). Plot (Figure 4A) illustrates individual gene intensity values (listed in this table) of HBMEC versus i-BEC for all genes assessed on the microarray. Genes showed significant differences between i-BEC and HBMEC are listed in Figure 4B.

Table 5. Reagents for iPSC differentiation protocols

**AFC/iPSC Reagents**
• DMEM Cat # 11965-092 (Life Technologies)<br>• FBS Cat # 080150 lot# 115651 (Wisent)<br>• DMEM/F12 Cat #10565-018 (Life Technologies)<br>• Corning Matrigel hESC Quality Cat # 354277 (Corning)<br>• Y27632 ROCK Inhibitor Cat # 72302 (Stem Cell Technologies)<br>• mTeSR1 Cat # 05850 (Stem Cell Technologies)<br>• ReLeSR Cat # 05872 (Stem Cell Technologies)<br>• DMSO Cat # D8418 (Sigma)<br>• StainAlive Dylight 488 Mouse anti-human TRA1-81 Antibody Cat # 09-0069 (Stemgent)
**Neural Differentiation**
• STEMdiff Neural Induction Medium Cat# 05835 (Stem Cell Technologies)<br>• STEMdiff Neural Progenitor medium Cat# 05833 (Stem Cell Technologies)<br>• Accutase Cat # 7920 (Stem Cell Technologies)<br>• Knock-out DMEM/F12 Cat #12660012 (Gibco)<br>• Accutase Cat # 7920 (Stem Cell Technologies)<br>• NPMM (Neural Progenitor Maintainance BulletKit Medium-Cat. CC3209 Lonza)<br>• B27 supplement (50X) Cat # 17504-044 (Life Technologies)<br>• N2 supplement (100X) Cat # 17502-048 (Life Technologies)<br>• Poly-L-ornithine Cat # P4957 (Sigma)<br>• Laminin Cat# 23017-015 (Gibco)<br>• Poly-L-lysine Cat# P4704 (Sigma)<br>• CTNF Cat # C-3835 (Sigma-Aldrich)<br>• Human recombinant EGF Cat # PHG-0311 (Life Technologies)<br>• Corning Matrigel hESC Quality Cat # 354277 (Corning)

**i-BEC Differentiation**
• mTeSR1 Cat # 05850 (Stem Cell Technologies)<br>• Accutase Cat # 7920 (Stem Cell Technologies)<br>• Knockout DMEM/F12 Cat # 12660-012 (Life Technologies)<br>• Knockout serum replacement Cat #10828-028 (Life Technologies)<br>• 100X MEM Non-Essential Amino Acids Solution Cat # 11140-050 (Life Technologies)<br>• Glutamax (100X) Cat # 35050-061 (Life Technologies)<br>• 2-Mercaptoethanol, 1000x solution Cat# 21985-023 (Life Technologies)<br>• Human Endothelial-SFM Cat # 11111-044 (Life Technologies)<br>• Platelet-Poor Plasma Derived Serum Cat # BT-214 (Alfa-Aesar)<br>• All-trans Retinoic Acid Cat # R2625 (Sigma)
**Transwell Plating**
• Gelatin from porcine skin Cat# G1890 (Sigma)<br>• 12 well companion plate Cat # 353503 (Falcon)<br>• 12 well cell culture insert, PET, 1 µm pore size Cat. # 353103 (BD-Falcon)<br>• Rat-tail Collagen Type 1 Cat # 354236 (VWR)<br>• Collagen IV Cat # C5533 (Sigma)<br>• Fibronectin Cat # F1056-1MG (Sigma)

Supplementary Figures

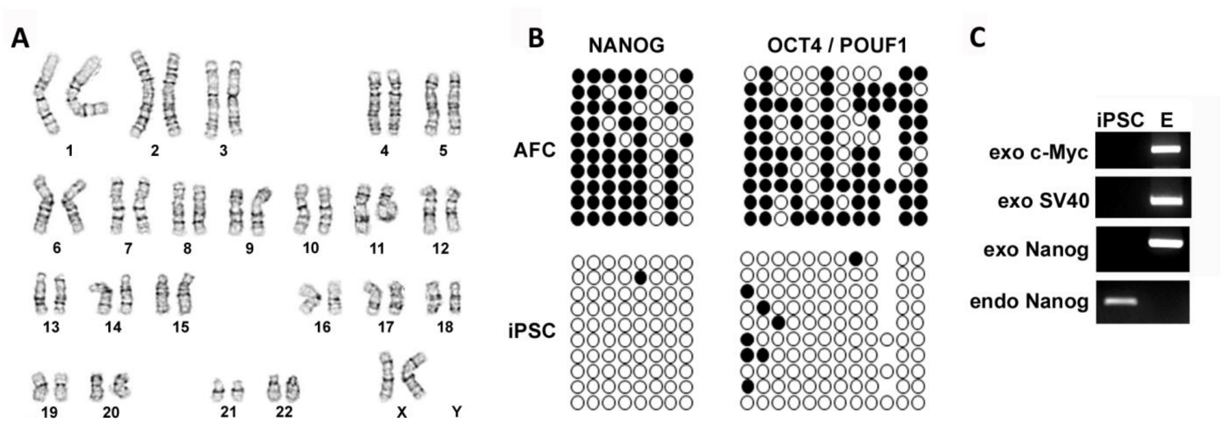

Supplementary Figure 1. Karyotpic stability and hypomethylation of NANOG and OCT4 promoters in AF-iPSCs.

(**A**). Representative karyotype analysis of AF-iPSCs based on 20 G-banding metaphase cells demonstrating normal chromosomes. (**B**) Analysis of the methylation status of the *OCT4* and *NANOG* promoters in AFC and iPSC using bisulfite sequencing. Open circles indicate unmethylated and closed circles indicate methylated CpG dinucleotides. (**C**) PCR analysis for episomal DNA in AF-iPSCs based on the expression of exogenous (exo) *c-Myc*, *SV40* and *NANOG* and endogenous (endo) *NANOG* expression to confirm exogenous episomal gene silencing. Cropped gels shown; Episomal DNA template (E) was used as positive control.

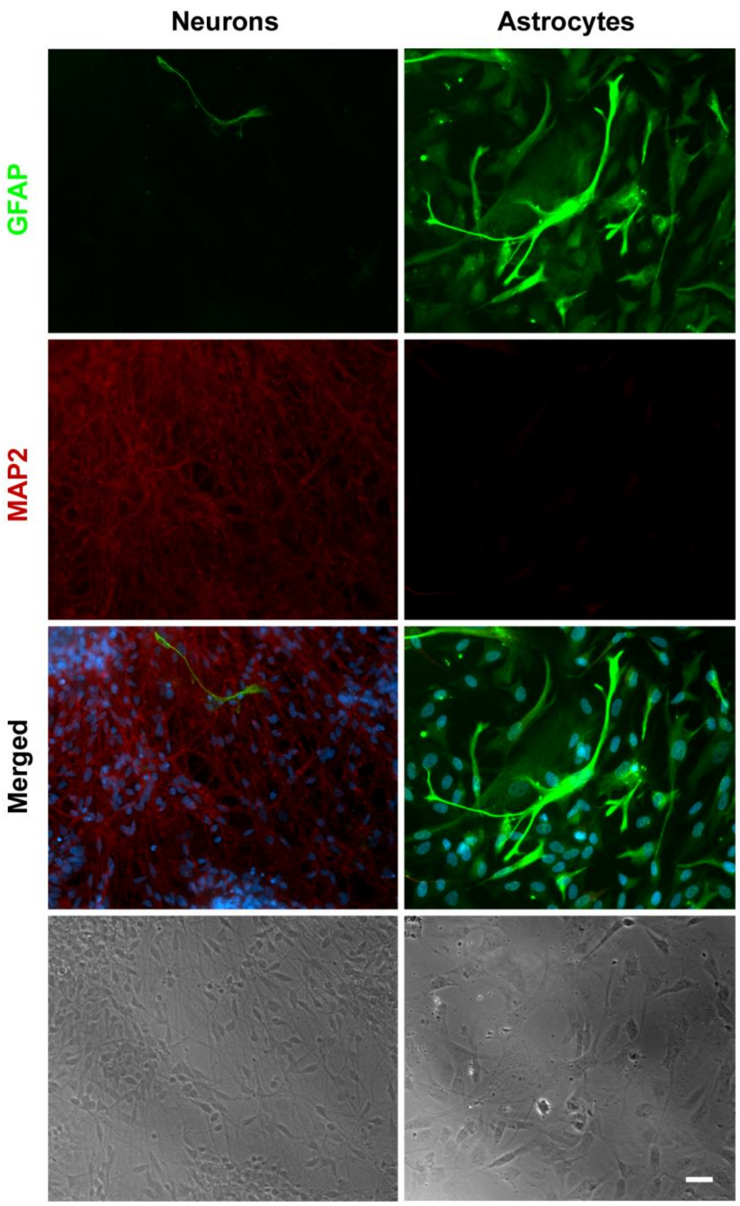

Supplementary Figure 2. Purity of AF-iPSC-derived neurons and astrocytes

Representative immunofluorescence confirming purity of iPSC-derived terminally differentiated neurons (MAP2) and astrocytes (GFAP). Note a single GFAP positive contaminating astrocyte

in neuronal cultures. No MAP2$^+$/GFAP$^+$ cells were observed confirming efficiency in differentiation. Nuclei counterstained with Hoescht. Scale bar, 20 µm.

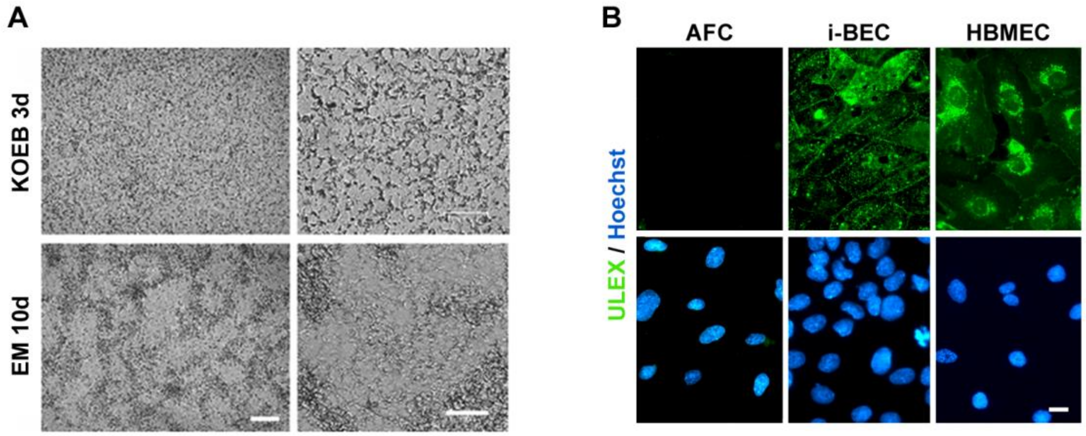

Supplementary Figure 3. Morphological changes of AF- iPSC cultures in KOEB and EM medium

(**A**) Representative phase contrast images showing morphological changes following the transition of AF-iPSC from mTesR1 medium to low-osmolality KnockOut DMEM/F12 medium (KOEB) and EM. Note homogeneity in 3 day (3D) KOEB culture at low magnification (left panel) and endothelial-like cobblestone morphology at higher magnification (right panel). Characteristic endothelial cobblestone morphology becomes more prominent following maturation for 10 days in EM. Scale bar, 100 µm (left panel) and 60 µm (right panel). (**B**) i-BECs are positive for ULEX staining, a lectin that selectively binds to the L-fucose residues in endothelial cells. HBMEC and AFCs were used as a positive and negative control, respectively. i-BECs ULEX staining was performed at 10 days in EM. Nuclei counterstained with Hoechst. Scale bar, 20 µm.

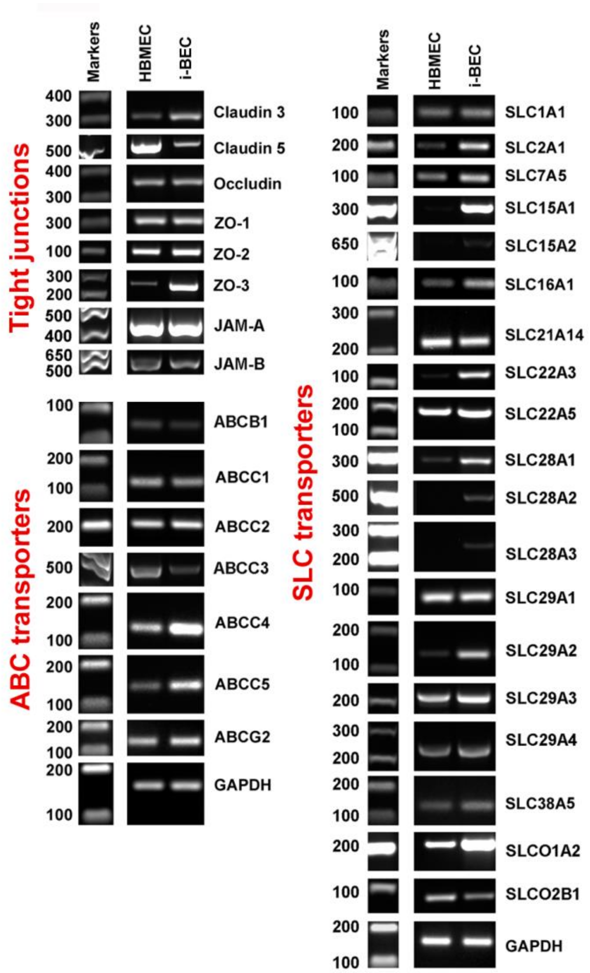

Supplementary Figure 4. RT-PCT transcript expression in i-BEC and HBMECs

Transcript expression characteristic of brain endothelial cells, such as tight junctions, ABC and SLC transporters examined by RT-PCR. Cropped gels shown; *GAPDH* and HBMEC were used as an internal and positive control, respectively. Primers listed in Supplementary Table 2.

μm.

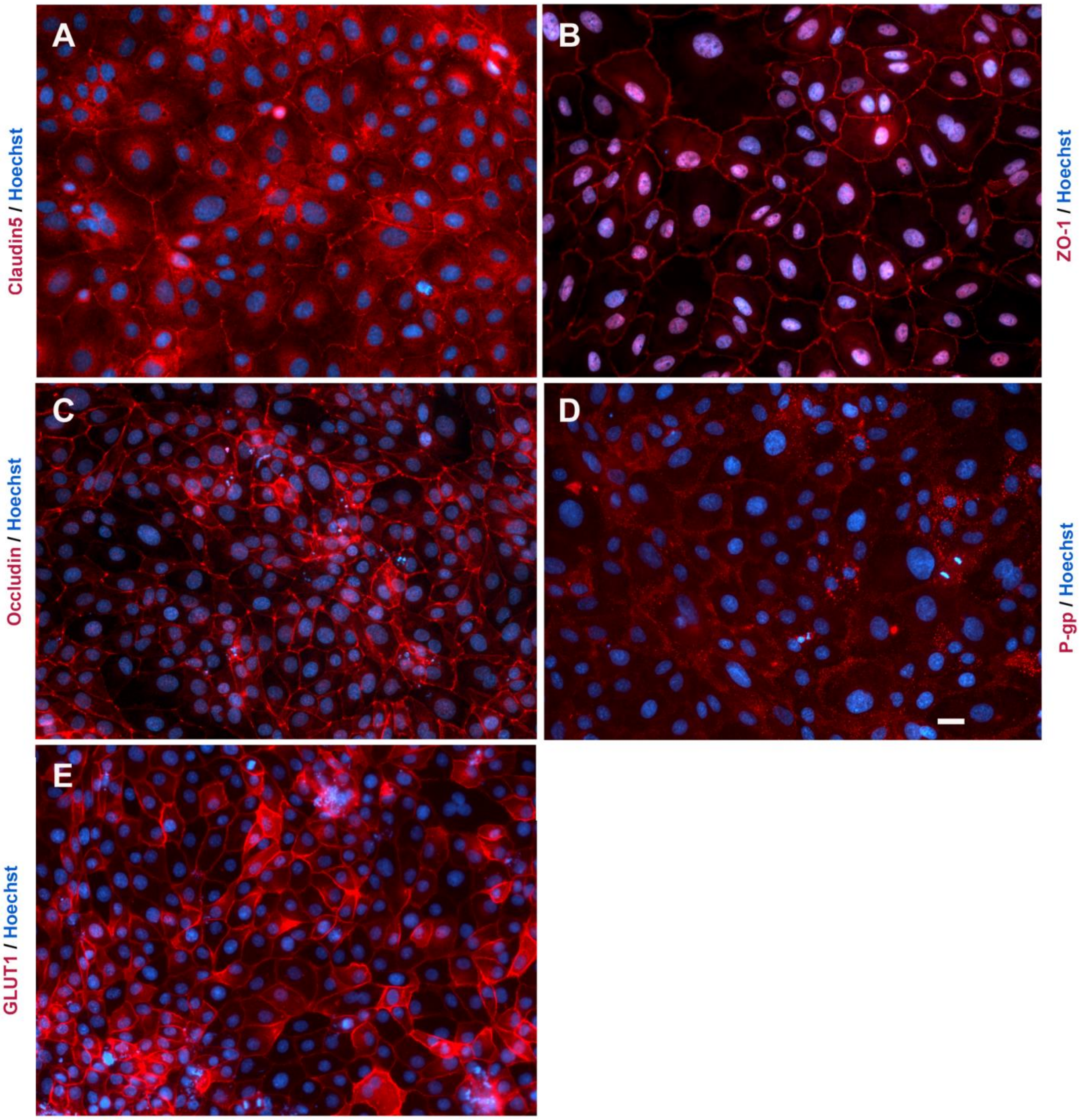

Supplementary Figure 5. Immunofluorescence images showing expression of requisite BBB markers

Low magnification immunofluorescence images depicting expression of requisite BBB markers Claudin-5, Occludin, GLUT1, ZO-1 and P-gp. Nuclei counterstained with Hoechst. Scale bar, 20 μm.

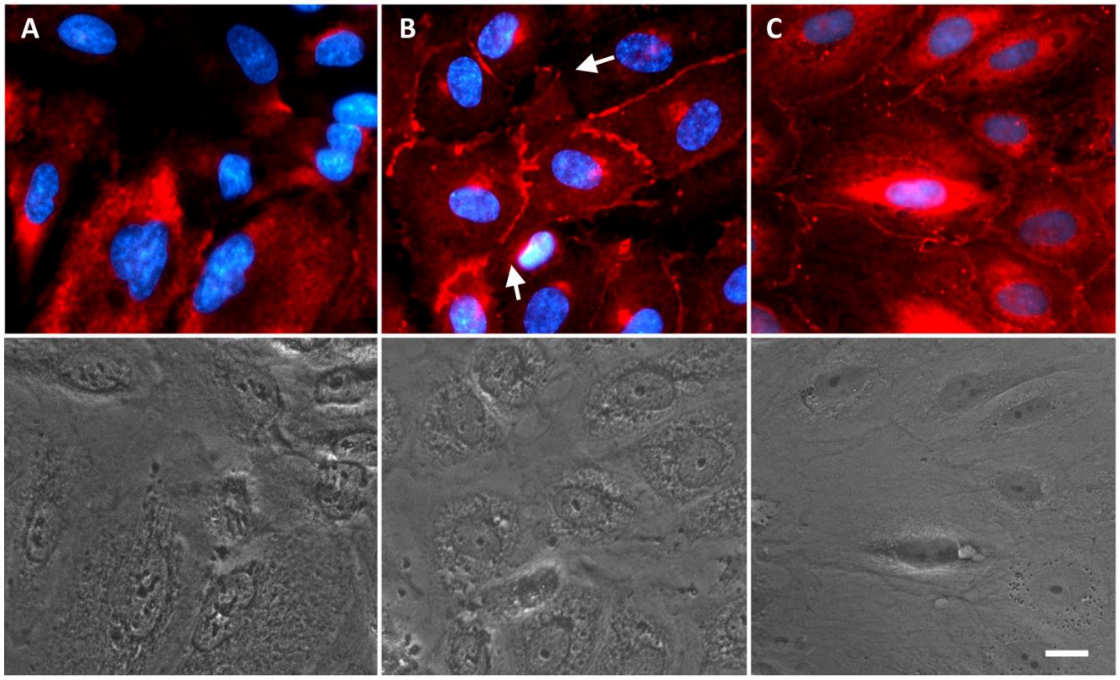

Supplementary Figure 6. Immunostaining depicting localization of Claudin-5 tight junctions in AFC, HBMEC and i-BECs.

Immunostaining of Claudin-5 showing diffuse, cytoplasmic localization in AFC (A) and discontinuous intercellular tight junctions in HBMEC cultures (B, arrow) compared to the continuous intercellular contacts in i-BECs (C). Nuclei counterstained with Hoechst. Scale bar, 20 µm.

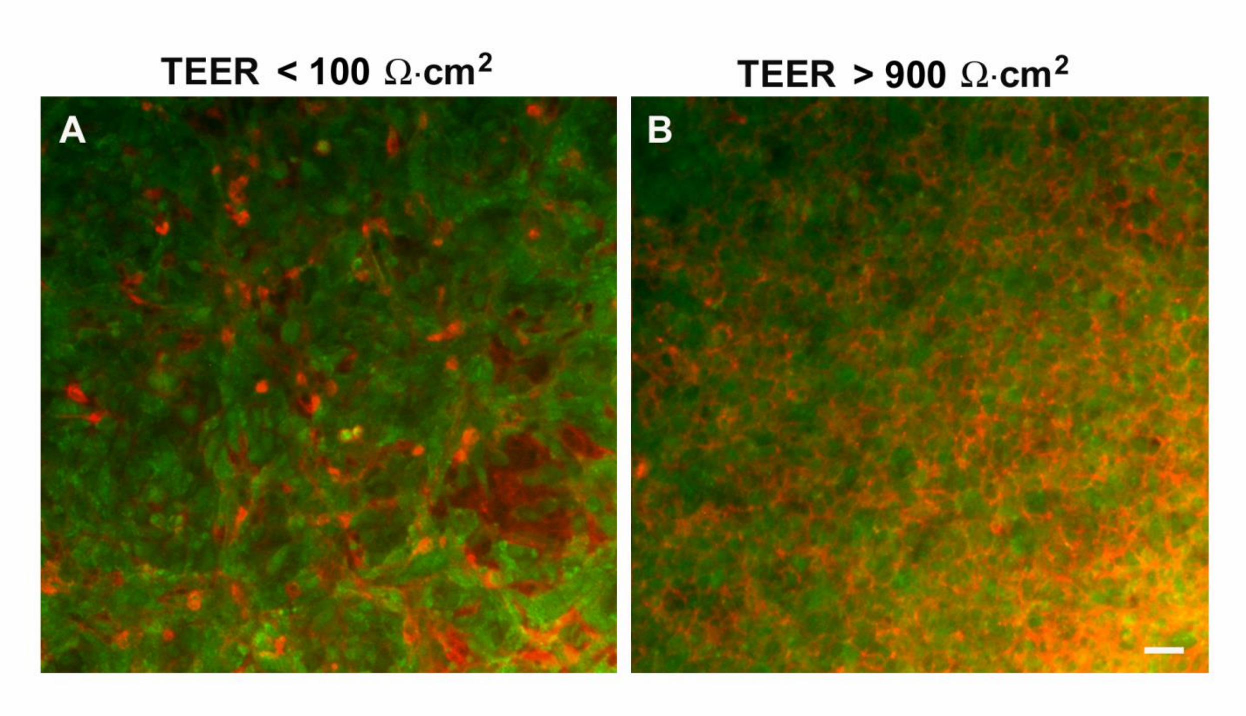

Supplementary Figure 7. CFDA/CMO staining depicting monolayer integrity

Representative lower magnification CFDA (green) and CMO (red) staining of i-BEC monolayers with accompanying TEER values. (**A**) Note visible intercellular spaces (devoid of CFDA staining) between adjacent i-BEC cells and lack of CMO positive cobblestone morphology on cultures that yield low TEER ($\leq$100 Ωcm^2). (**B**) Tight packing of i-BEC cells with continuous membrane contacts and no visible intercellular gaps reflected in high TEER ($\geq$100 Ωcm^2). Scale bar, 40 µm.

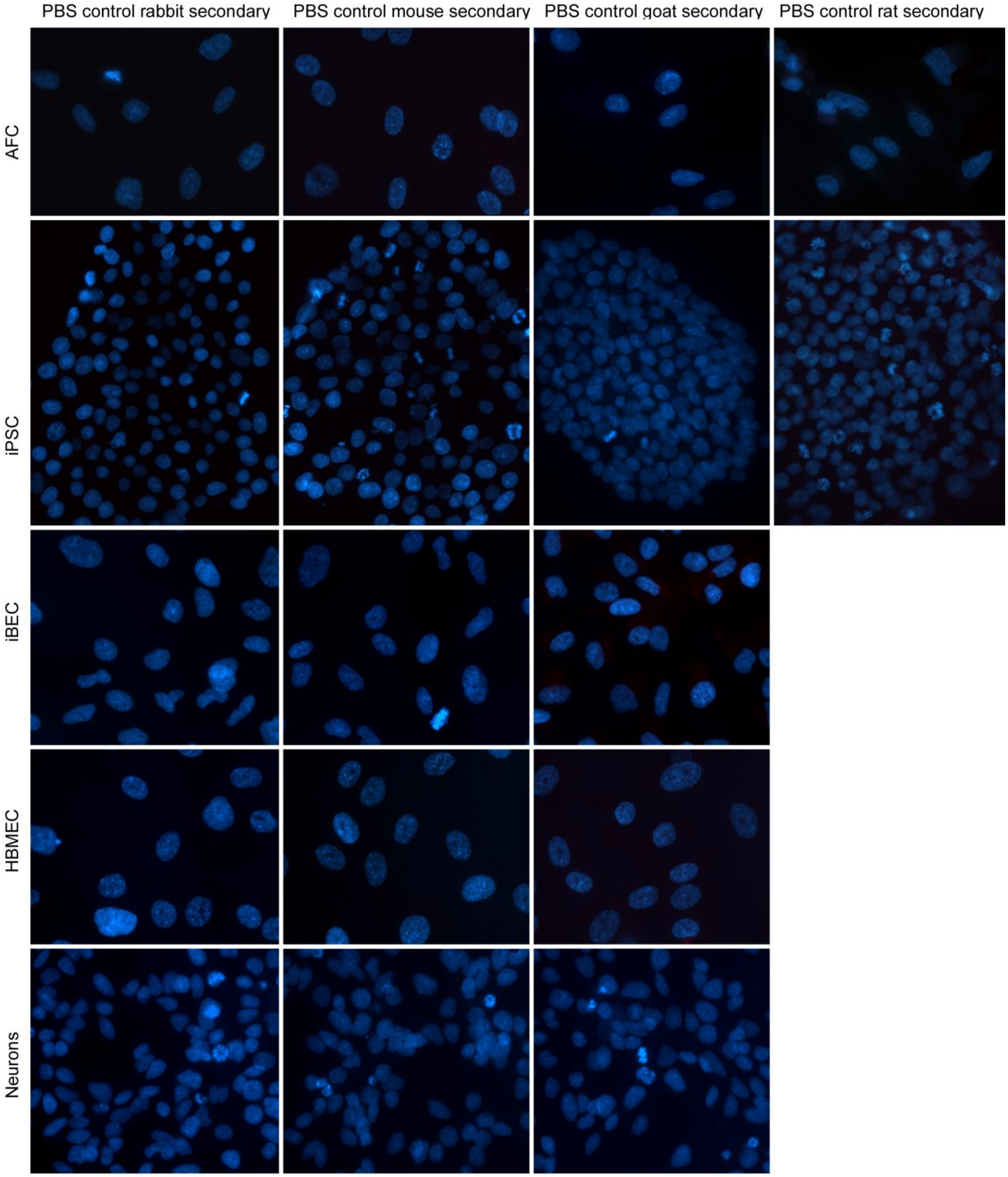
PBS control rabbit secondary
PBS control mouse secondary
PBS control goat secondary
PBS control rat secondary
AFC
iPSC
iBEC
HBMEC
Neurons

Supplementary Figure 8. Negative controls for immunstaining.

PBS only secondary controls are presented, overlaid with Hoechst, for each cell line studied and secondary antibody used.

Full length western blots (Figure 1 and Figure 2)

ACTIN (Figure 1)

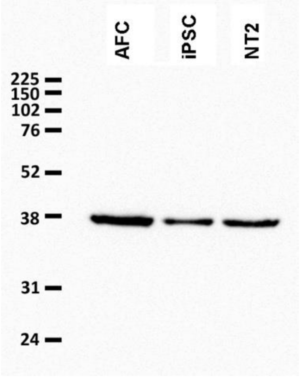

KLF4 (Figure 1)

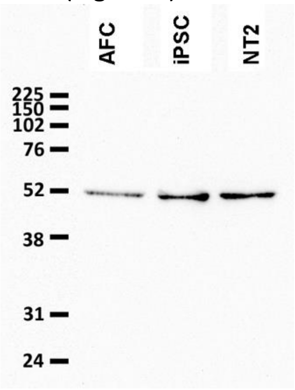

NANOG (Figure 1)

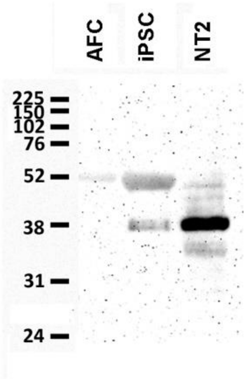

SOX2 (Figure 1)

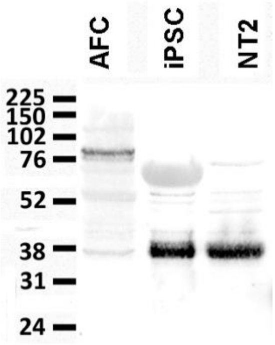

PAX6 (Figure 2)

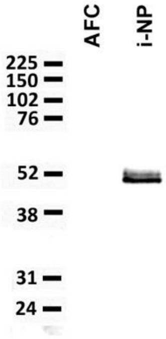

NESTIN (Figure 2)

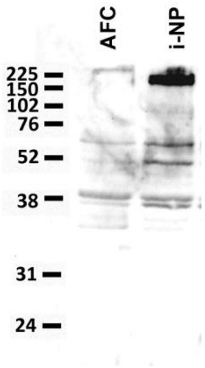

SOX2 (Figure 2)

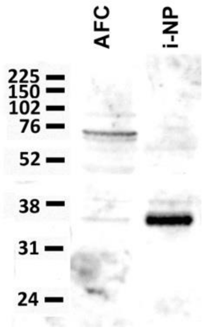

Full length RT-PCR gels

Lane Annotation**: 1:HBMEC, 2: iBEC, 3: NT2**

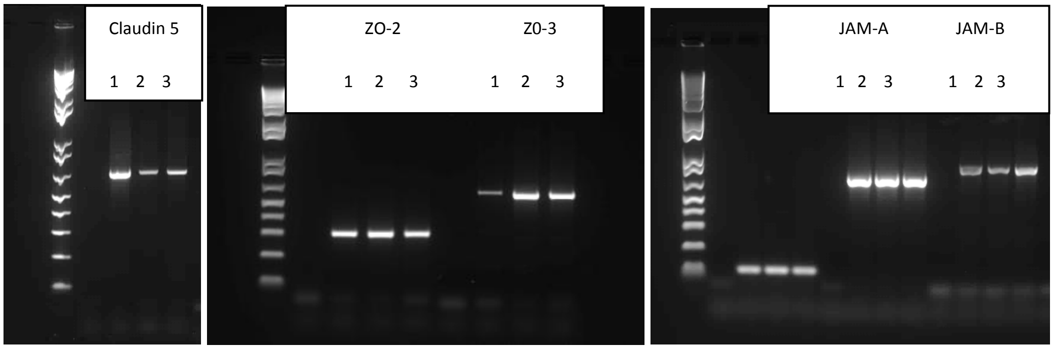
Claudin 5
1 2 3
ZO-2
1 2 3
Z0-3
1 2 3
JAM-A
1 2 3
JAM-B
1 2 3

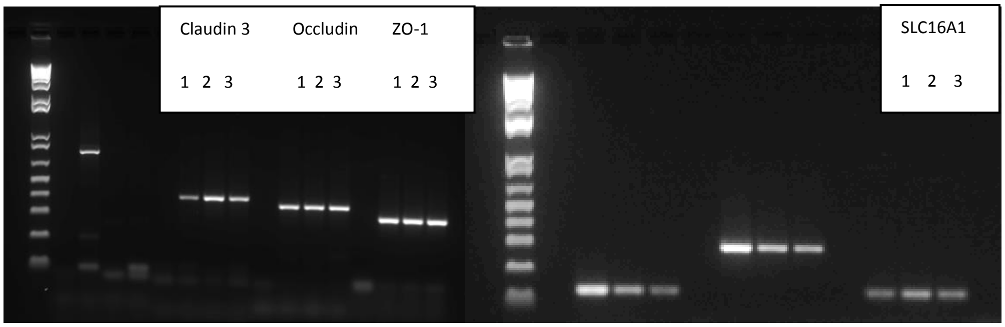
Claudin 3
1 2 3
Occludin
1 2 3
ZO-1
1 2 3
SLC16A1
1 2 3

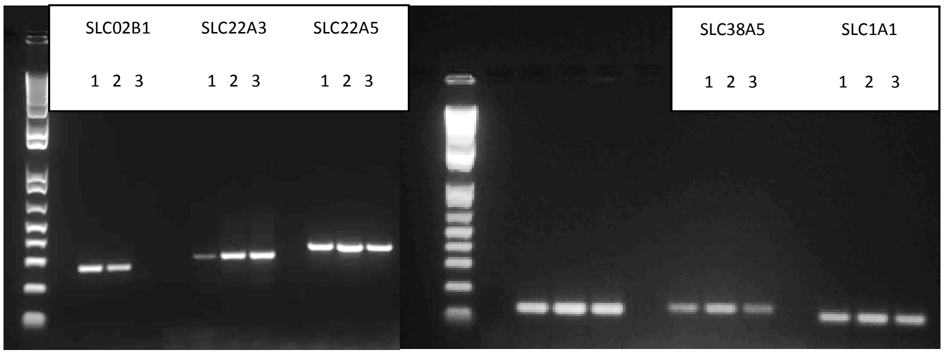
SLC02B1
1 2 3
SLC22A3
1 2 3
SLC22A5
1 2 3
SLC38A5
1 2 3
SLC1A1
1 2 3

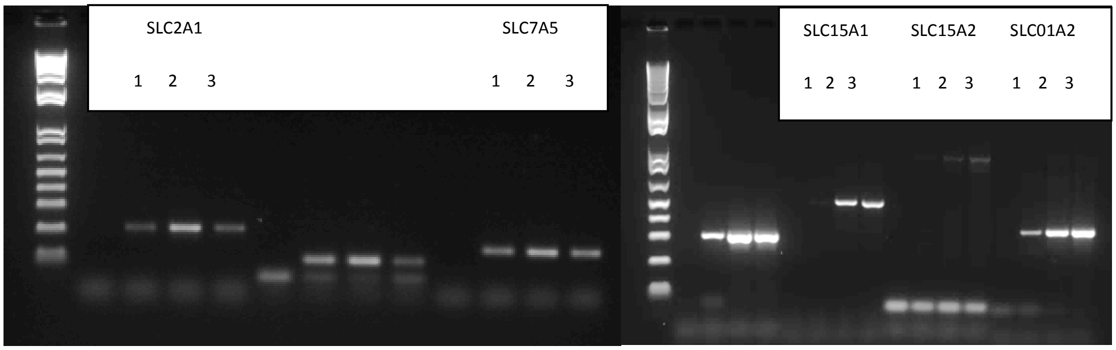
SLC2A1
1 2 3
SLC7A5
1 2 3
SLC15A1
1 2 3
SLC15A2
1 2 3
SLC01A2
1 2 3

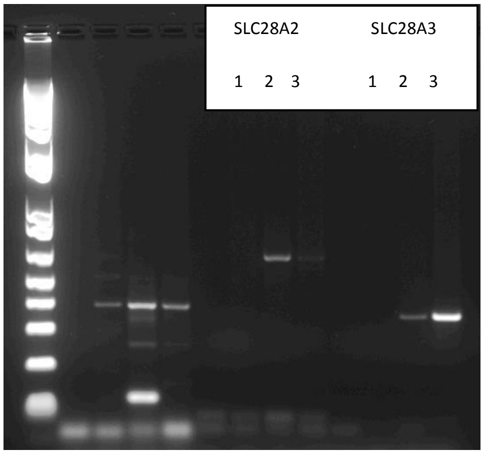
SLC28A2
SLC28A3
1 2 3
1 2 3
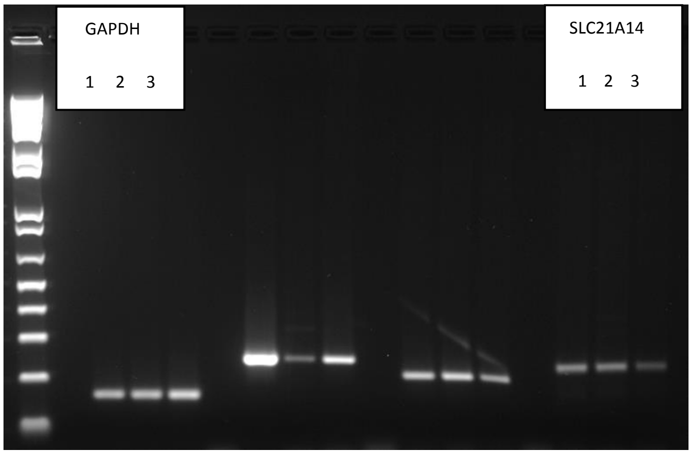
GAPDH
1 2 3
SLC21A14
1 2 3
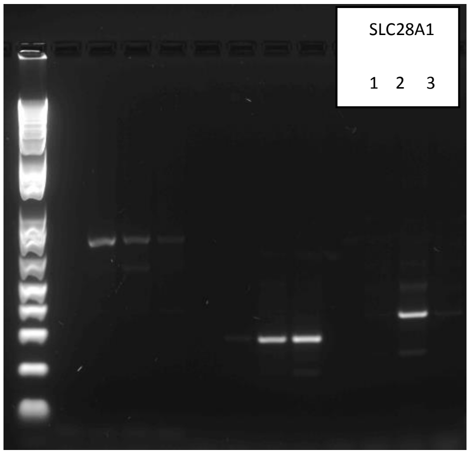
SLC28A1
1 2 3
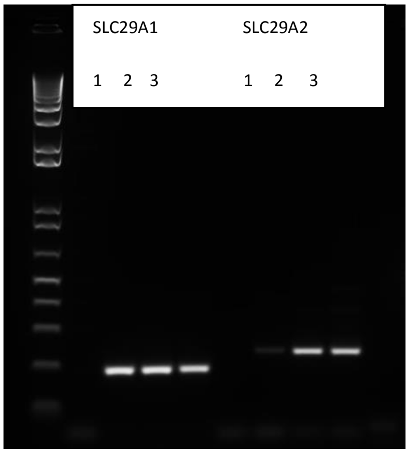
SLC29A1
SLC29A2
1 2 3
1 2 3

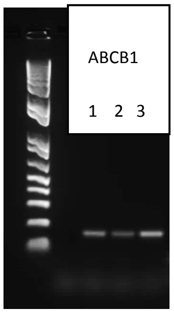
ABCB1
1 2 3

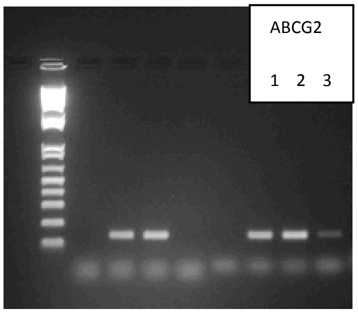
ABCG2
1 2 3

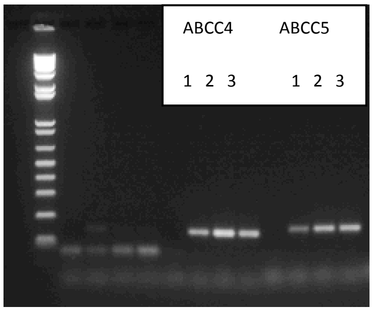
ABCC4
1 2 3
ABCC5
1 2 3

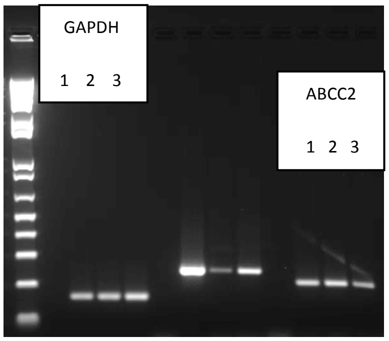
GAPDH
1 2 3
ABCC2
1 2 3

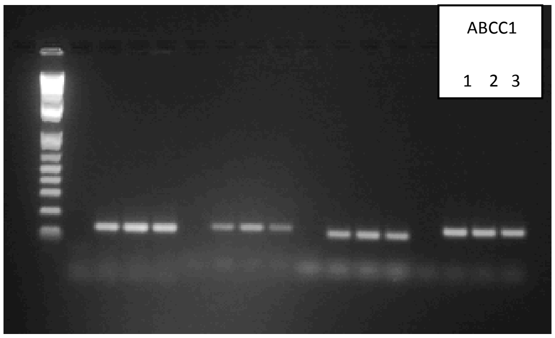
ABCC1
1 2 3

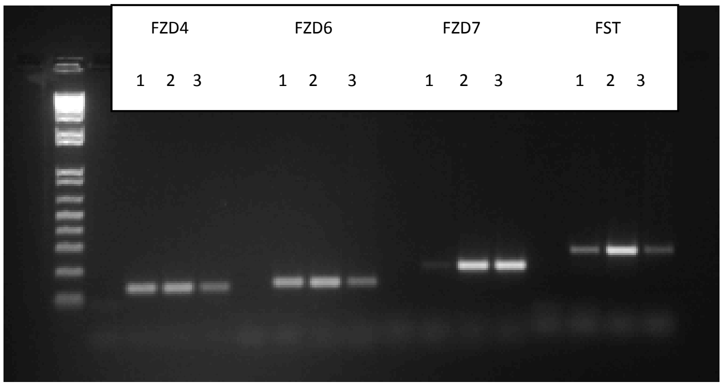
FZD4
1 2 3
FZD6
1 2 3
FZD7
1 2 3
FST
1 2 3

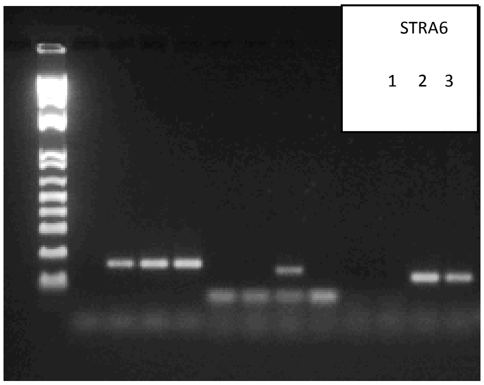
STRA6
1 2 3

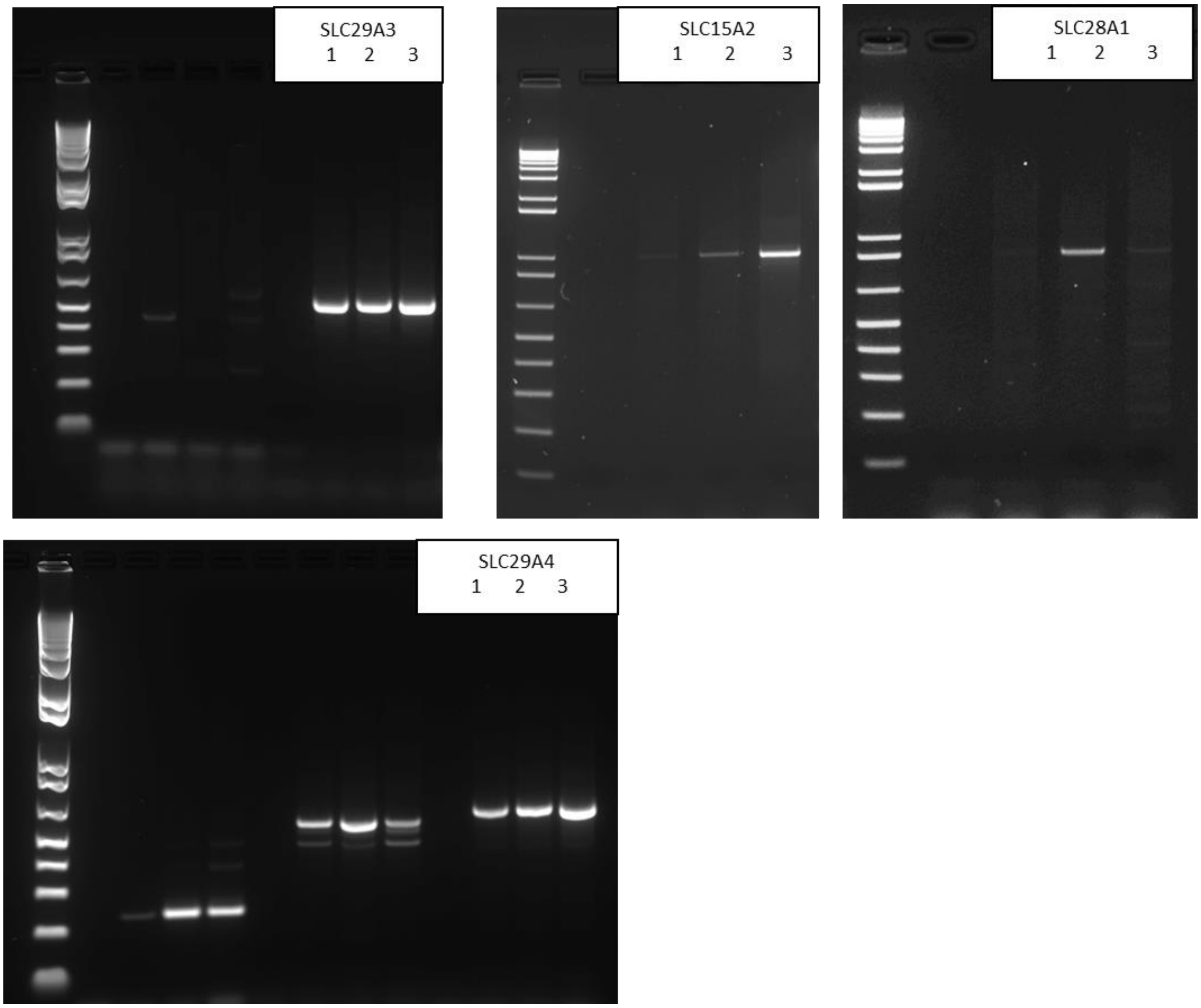
SLC29A3
1 2 3
SLC15A2
1 2 3
SLC28A1
1 2 3
SLC29A4
1 2 3
